# Prior knowledge changes initial sensory processing in the human spinal cord

**DOI:** 10.1126/sciadv.adl5602

**Published:** 2025-01-15

**Authors:** Max-Philipp Stenner, Cindy Márquez Nossa, Tino Zaehle, Elena Azañón, Hans-Jochen Heinze, Matthias Deliano, Lars Büntjen

**Affiliations:** ^1^ Leibniz Institute for Neurobiology Magdeburg, Magdeburg, Germany.; ^2^ Department of Neurology, Otto-von-Guericke University Magdeburg, Magdeburg, Germany.; ^3^ Center for Behavioral Brain Sciences Magdeburg, Magdeburg, Germany.; ^4^ Center for Intervention and Research on adaptive and Maladaptive Brain Circuits Underlying Mental Health, Jena-Magdeburg-Halle, Magdeburg, Germany.; ^5^ Department of Neurosurgery, Otto-von-Guericke University Magdeburg, Magdeburg, Germany.; ^6^ Department of Stereotactic Neurosurgery, Otto-von-Guericke University Magdeburg, Magdeburg, Germany.

## Abstract

Prior knowledge changes how the brain processes sensory input. Whether knowledge influences initial sensory processing upstream of the brain, in the spinal cord, is unknown. Studying electric potentials recorded invasively and noninvasively from the human spinal cord at millisecond resolution, we find that the cord generates electric potentials at 600 hertz that are modulated by prior knowledge about the time of sensory input, as early as 13 to 16 milliseconds after stimulation. Our results reveal that already in the spinal cord, sensory processing is under top-down, cognitive control, and that 600-hertz signals, which have been identified as a macroscopic marker of population spiking in other regions of the nervous system, play a role in early, context-dependent sensory processing. The possibility to examine these signals noninvasively in humans opens up avenues for research into the physiology of the spinal cord and its interaction with the brain.

## Teaser

High-frequency signals at a neuronal population level reveal a cognitive influence on sensory processing in the human spinal cord.

## INTRODUCTION

We perceive the world in light of our prior experience. Prior knowledge can help suppress redundant information (*1*, *2*) and improve the quality of sensory representation (*3*, *4*). Given its fundamental role in contemporary theories of perception (*5*), a key question is whether prior knowledge modulates sensory processing already at initial stages (*2*).

For somatosensory input, the earliest stage of processing in the central nervous system is the spinal cord. Here, we ask whether prior knowledge modulates the initial processing of somatosensory input in the human spinal cord.

At a population level, neurons in the human spinal cord generate two types of signals in response to sensory input. Besides classic short-latency evoked responses (*6*, *7*), sensory input induces high-frequency signals at around 600 Hz. High-frequency signals have been described in cortical and subcortical regions of the somatosensory system in the brain (*8*), and, recently, also in the human spinal cord (*9*, *10*). However, despite their prevalence throughout the somatosensory system, the functional role of high-frequency signals in sensory processing has remained enigmatic.

Phase-locking of single-unit activity to macroscopic 600-Hz signals in nonhuman primates indicates that signals at 600 Hz represent a macroscopic marker of spiking, i.e., neuronal output, at least at the level of cortex (*11*, *12*). Hence, they carry information that is complementary to low-frequency signals (*9*, *13*), including event-related potentials (ERPs), which predominantly reflect mass synaptic input (*14*). Previous studies have proposed that high-frequency signals may reflect a local network state (*13*), possibly set by factors that modulate perception, such as attention (*15*). However, evidence for an involvement of 600-Hz signals in top-down modulation has remained scarce.

**Fig. 1.**
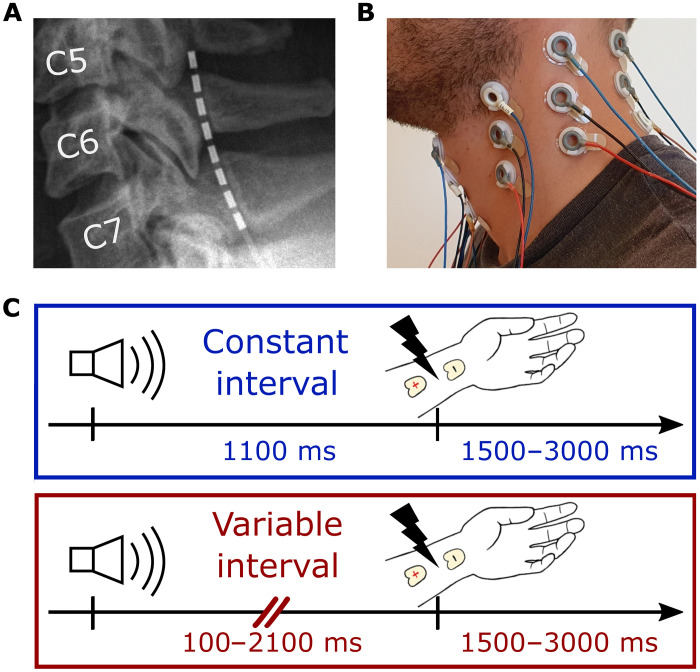
Electrode placement for invasive and noninvasive recordings and schematic of the experimental task. (**A**) Invasive recordings, (**B**) noninvasive recordings, and (**C**) task schematic. A total of 1558 trials were completed per condition, on average, in the noninvasive study, while patients completed, on average, 338 trials per condition during invasive recordings.

Here, we examined whether 600-Hz signals are involved in top-down control at initial stages of sensory processing, as early as in the spinal cord. We recorded electric potentials invasively from eight electrodes on an epidural lead inserted into the spinal canal alongside the dorsal cervical spinal cord (Fig. 1A) in eight patients with neuropathic pain undergoing effectiveness testing of electric spinal cord stimulation for pain relief (see fig. S1A and table S1). In addition, we confirmed results obtained in patients by recording electric potentials noninvasively in 30 healthy subjects, via an array of 24 electroencephalography (EEG) electrodes placed around the neck (Fig. 1B).

In both groups, we examined the spinal cord response to electric median nerve stimulation in two conditions (Fig. 1C, within-subject design). In both conditions, an auditory cue preceded median nerve stimulation. In one condition, the time interval between the cue and median nerve stimulation was constant (1100 ms; constant-interval condition), allowing subjects to precisely predict when median nerve stimulation would be delivered. In the other condition (variable-interval condition), the interval between the auditory cue and median nerve stimulation varied randomly from trial to trial, between 100 and 2100 ms (uniform distribution, mean = 1100 ms), preventing precise temporal prediction of median nerve stimulation. In both conditions, subjects silently counted the number of rare targets in each block. Targets consisted of a rapid succession of two median nerve stimulations separated by 80 ms. Of the 100 trials in each block, between 5 and 10 contained a target. At the end of each block, subjects reported the number of targets in that block.

## RESULTS

In line with the idea that temporal predictability enhances perception (*3*), healthy subjects were significantly more accurate in counting targets in the constant-interval condition compared to the variable-interval condition [*t*(29) = 2.4, *P* = 0.024, and *d* = 0.44, proportion of blocks for which subjects counted correctly: 68.2 ± 19.2% versus 62.9 ± 21.5%, mean ± SD]. For the patients, there was a trend in the same direction [*t*(7) = 2.36, *P* = 0.05, and *d* = 0.84, 60.62 ± 43.04% versus 38.54 ± 39.29%; fig. S2].

**Fig. 2.**
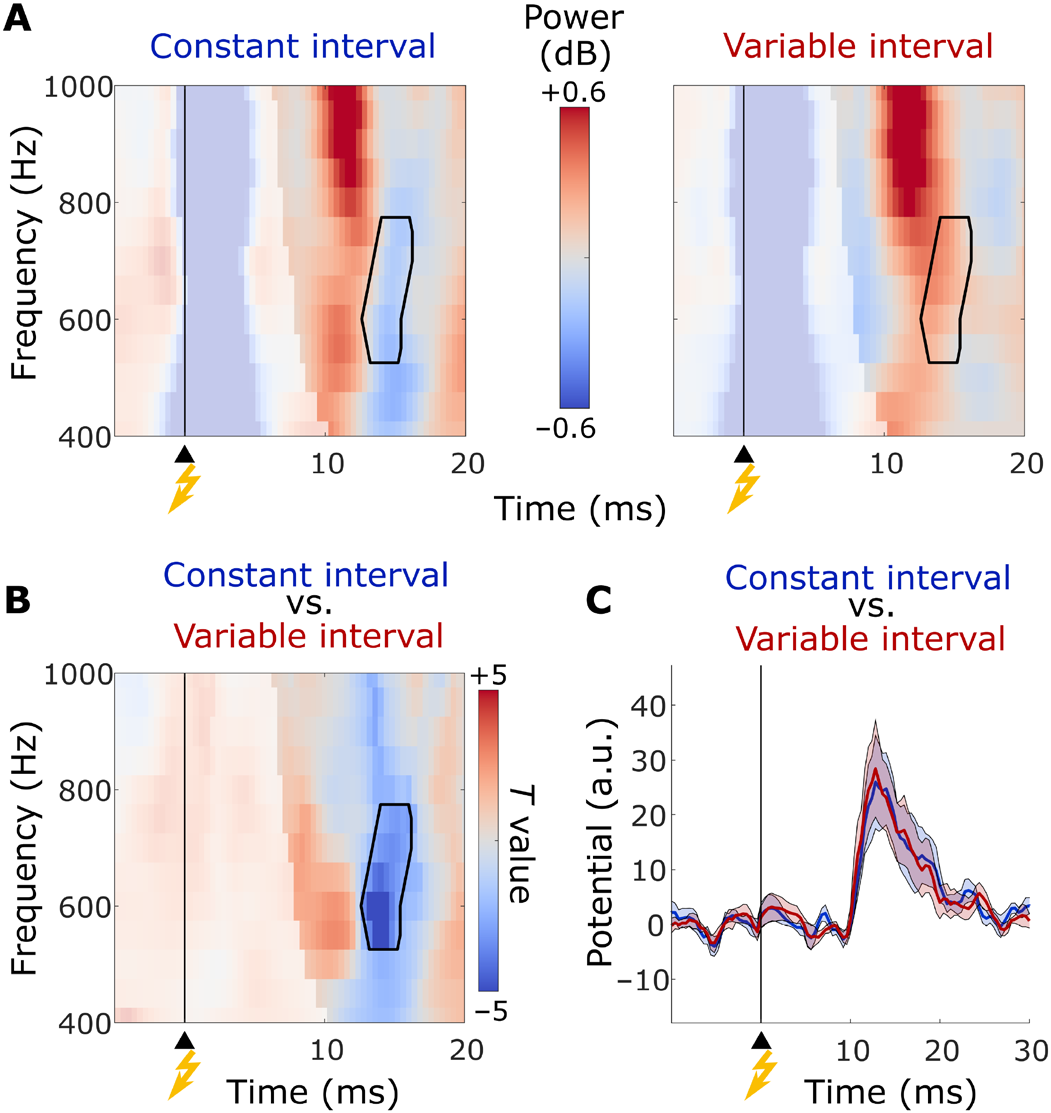
Time- and frequency-resolved power, and grand-average evoked response, in the constant- and variable-interval conditions for invasive recordings. (**A**) Time- and frequency-resolved power. (**B**) *T* values for condition differences in power. (**C**) Evoked response in the constant-interval condition (blue) and variable-interval condition (red). (A) and (C) show grand means, and (B) shows *T* values at the group level. The contour in (A) and (B) represents the spectrotemporal extent of the cluster that survived permutation testing. Time-frequency bins possibly affected by the stimulation artifact, given the frequency-dependent taper (five cycles), are semi-opaque. The shading in (C) represents SEM. In all panels, the yellow flash on the *x* axis indicates the time of median nerve stimulation.

To examine whether knowledge about the time of stimulation modulates initial sensory processing in the spinal cord, we compared electric potentials between conditions, in an a priori defined time window between 8 and 16 ms after median nerve stimulation (*9*). To account for interpatient variation in electrode location alongside the cervical spinal cord, we used spatial filtering (*16*) to extract, for each individual, a virtual channel that maximized the evoked response to median nerve stimulation (fig. S1). Besides the evoked response, median nerve stimulation induced an increase in high-frequency power above baseline within the first 16 ms (Fig. 2A), confirming previous observations (*9*, *10*).

A cluster-based permutation test revealed that power between 200 and 1000 Hz, and in a time window between 8 and 16 ms, was significantly lower in amplitude in the constant-interval condition, compared to the variable-interval condition (*P* = 0.039 and *d* = 1.78; mean power ± SD in the identified cluster: −0.095 ± 0.284 dB versus 0.201 ± 0.297 dB, respectively; Fig. 2B). The amplitude of the evoked response, on the other hand, did not differ significantly between conditions (no clusters found between 8 and 16 ms; Fig. 2C), nor did phase-locked responses between 200 and 1000 Hz (*P* > 0.8; fig. S3, A and B). The difference in high-frequency power persisted after attenuating phase-locked components (figs. S4 and S5), and was robust to the choice of baseline (fig. S6, A and B). Prior knowledge about the time of median nerve stimulation therefore modulated phase-jittered, high-frequency signals induced by stimulation.

There was evidence that this modulation involved a shift in the latency of peak power, identified in the time window of interest (between 8 and 16 ms). In the constant-interval condition, high-frequency power, averaged between 400 and 800 Hz, peaked at 11 ± 1.3 ms (mean ± SD). This peak shifted to 13.5 ± 1.9 ms in the variable-interval condition [*t*(7) = 2.55, *P* = 0.038, and *d* = 0.9; similar results were obtained when averaging power between 550 and 750 Hz, i.e., the spectral extent of the cluster shown in Fig. 2B]. A jackknife-based estimation of onset latencies, defined using a relative criterion of 50% peak-power across conditions (*17*), confirmed a significant latency shift {from 8.9 ms in the constant-interval condition to 10.5 ms in the variable-interval condition [mean onset latency; *t*(7) = 2.44 and *P* = 0.045]}.

**Fig. 3.**
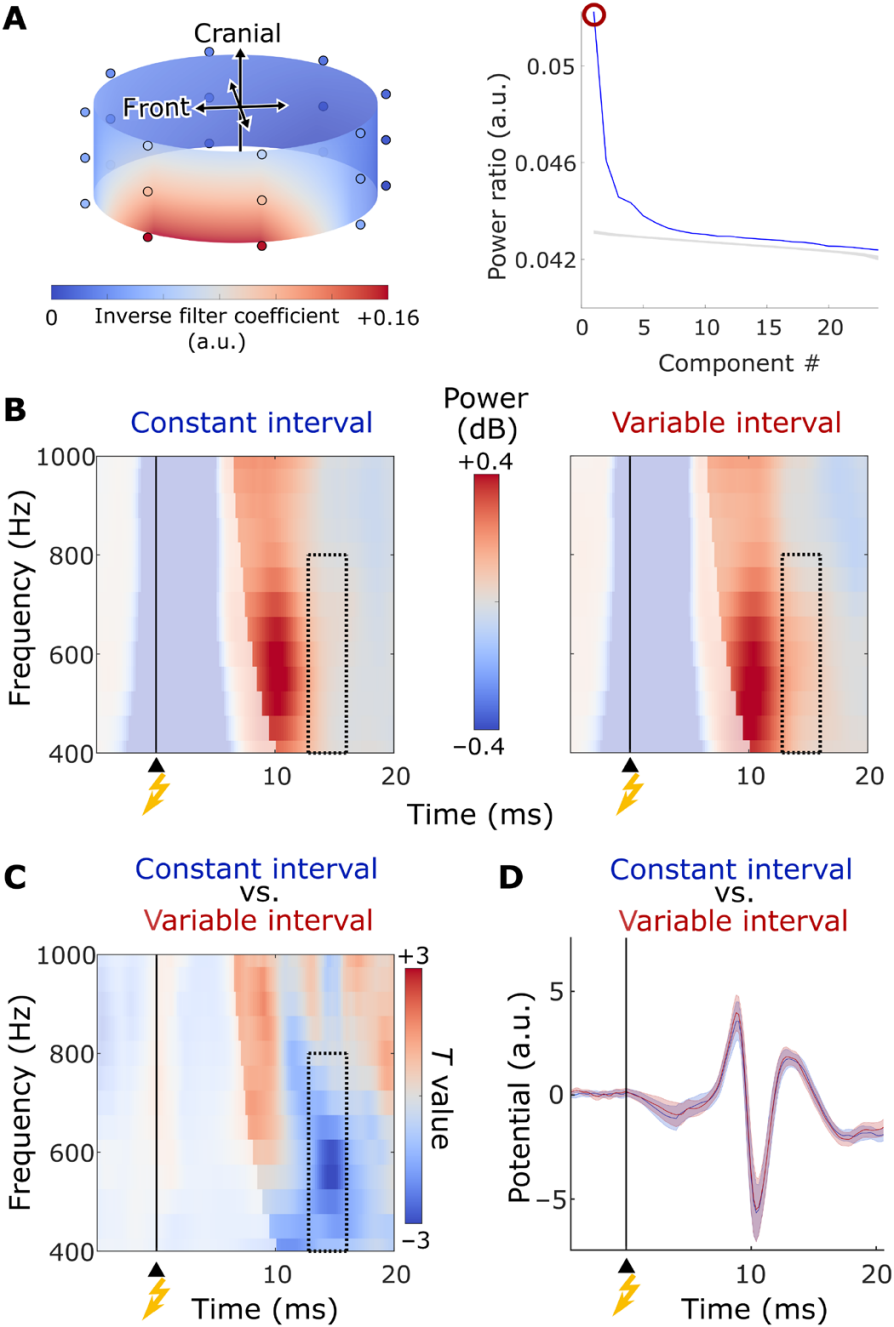
Time- and frequency-resolved power, and evoked response, in the constant- and variable-interval conditions for noninvasive recordings. (**A**) Left, spatial pattern of the first component of spatial filtering (inverse of the filter coefficients, displayed for each electrode location). Right, power ratio of all components (blue). The gray shading represents the 5 to 95% percentile range of power ratios obtained in a resampling test. We chose the first component (red circle), i.e., the component reflecting the strongest increase in 400 to 800 Hz power in our time window of interest. (**B**) Time- and frequency-resolved power, and (**C**) *T* values for condition differences in power. The contour represents the a priori defined time-frequency window used for the cluster-based permutation test, guided by results obtained from invasive recordings (400 to 800 Hz, and 13 to 16 ms). Time-frequency bins possibly affected by the stimulation artifact, given the frequency-dependent taper (five cycles), are semi-opaque. (B) and (C) show data after attenuating phase-locked components (see fig. S8 for complementary results without attenuating phase-locked responses). (**D**) Evoked response in the constant-interval condition (blue) and variable-interval condition (red). The shading represents SEM. (B) and (D) show grand means, and (C) shows *T* values at the group level. In (B), (C), and (D), the yellow flash on the *x* axis indicates the time of median nerve stimulation.

Electric potentials recorded from the surface of the neck of healthy subjects confirmed a difference in short-latency, high-frequency power between conditions. From the original 24 channels, spatial filtering (*9*, *16*) derived a single virtual channel that captured the stimulus-induced increase in high-frequency power (*9*, *10*) (Fig. 3A). We expected lower high-frequency power in the constant-interval condition, compared to the variable-interval condition, in a time window between 13 and 16 ms, and a frequency window between 400 and 800 Hz, based on our invasive recordings (Fig. 2). Furthermore, we expected this effect to persist when attenuating phase-locked responses. Our noninvasive data confirmed that the high-frequency response in this time-frequency window was significantly lower in the constant-interval condition, compared to the variable-interval condition, even when attenuating phase-locked responses (*P* = 0.009, cluster-based permutation test; *d* = 0.658; mean power ± SD in the identified cluster: 0.036 ± 0.176 dB versus 0.108 ± 0.191 dB, respectively; Fig. 3, B and C, and fig. S7). This difference was also present when phase-locked responses were not attenuated (fig. S8), and persisted irrespective of the choice of baseline (fig. S6, C and D). The evoked response, on the other hand, did not differ significantly between conditions (no clusters found between 13 and 16 ms; Fig. 3C and fig. S9), nor did phase-locked responses between 400 and 800 Hz (no corresponding clusters found; fig. S3, C and D). We confirmed that the condition difference in high-frequency power in the noninvasive data persisted when taking into account only those variable-interval trials for which the time interval between the auditory cue and median nerve stimulation fell within a range of 1100 ± 200 ms (i.e., similar to the interstimulus interval in the constant-interval condition; fig. S10). Unlike the invasive recordings, the noninvasive recordings did not reveal any significant latency shift [peak of mean power between 400 and 800 Hz: 10.5 ± 1.1 ms (constant-interval condition) versus 10.6 ± 1 ms (variable-interval condition), mean ± SD; *t*(29) = 0.87 and *P* = 0.39].

To ensure that the difference in short-latency, high-frequency power between conditions was not due to changes in muscle contraction on the stimulated arm, we conducted two control experiments (*n* = 16 healthy, naïve individuals). We observed no difference between the constant-interval condition and the variable-interval condition in electromyography activity on the stimulated arm (fig. S11). Furthermore, in a separate task, voluntary muscle contraction could not reproduce the pattern of high-frequency modulation by temporal predictability observed in our invasive and noninvasive study (fig. S12).

## DISCUSSION

The possibility that cognition modulates sensory processing already in the spinal cord has long been overlooked. With the development of protocols for functional imaging of the human spinal cord (*18*), and tools for cerebrospinal circuit analysis in rodents (*19*), there has been a recent surge of interest in the role of the spinal cord in context-dependent perception.

Nociceptive, thermal, and tactile stimuli are locally processed in microcircuits of the spinal dorsal horn (*20*). These microcircuits are candidate sites for modulation of synaptic transmission by descending pathways. In rodents, corticospinal projections originating from primary and secondary somatosensory cortex facilitate processing of light touch in the spinal cord dorsal horn and contribute to the pathophysiology of neuropathic pain (*19*). However, it has remained unclear under which conditions, and with which consequences, this pathway is recruited under healthy conditions, and whether a supraspinal modulation of early sensory processing in the spinal cord exists in humans.

In humans, spinal functional magnetic resonance imaging (fMRI) has revealed cognitive factors that modulate blood oxygenation in the dorsal horn in response to nociceptive input (*18*, *21*, *22*); however, the latency and domain generality of this modulation have remained unclear. Specifically, fMRI cannot reveal whether this modulation occurs during the initial processing of sensory input, or as a feedback response in the spinal cord to later-stage processing in the brain. It is also unclear whether the influence of knowledge on spinal sensory processing is specific to pain, or a general principle of spinal sensory processing, including innocuous sensory input, which is processed along distinct spinal pathways (*23*).

Our combined invasive and noninvasive, electrophysiological inquiry into the initial sensory processing in the human spinal cord fills these gaps. We find that the human spinal cord processes innocuous input as a function of prior knowledge about the time of stimulation. The spinal cord thus participates in the context-dependent processing of sensory input that allows us to interpret our environment.

This opens up avenues for understanding how mental processes modulate initial sensory processing in the spinal cord and the underlying cerebrospinal circuitry. The short latency of the observed modulation by prior knowledge suggests a state change to the spinal sensory system already before the time of median nerve stimulation, and therefore a “tonic,” sustained top-down effect throughout at least part of the time interval between the cue and median nerve stimulation. This state change becomes detectable in the presence of afferent drive, when the large signals induced by median nerve stimulation reveal state-dependent processing, evident in the high-frequency response.

In our experimental paradigm, knowledge about the time of stimulation allows for temporal expectation, as well as temporally focused attention. Both would be consistent with the observed direction of modulation, i.e., a reduction in signal amplitude for expected, or efficiently attended, stimuli, compared to relatively unexpected, less efficiently attended stimuli. Expectation suppression is a typical finding across sensory systems, and often interpreted as the result of a filtering process that “explains away” expected information, reducing redundancy (*2*). On the other hand, expectation, like attention, may also sharpen representations by reducing noise contributions from populations of neurons that do not code for the expected or attended features (*3*). This, too, would result in reduced signal amplitudes at the level of neuronal population signals. Future work may use experimental paradigms that dissociate between expectation and attention (*24*) and examine the underlying circuitry, e.g., via simultaneous cerebrospinal fMRI (*25*).

Our findings highlight an important role of 600-Hz responses to sensory input as a macroscopic marker of spinal sensory processing under top-down control. Independent spontaneous amplitude fluctuations recorded at the scalp (*13*, *15*), and from the spinal cord (*9*), indicate that high-frequency signals carry unique information that is complementary to information carried by the ERP. There has been speculation about the mental processes underlying these fluctuations (*9*, *15*); however, the unique information content carried by high-frequency signals and their role in perception have remained elusive. We demonstrate that 600-Hz signals are sensitive to prior knowledge.

The cellular substrates of 600-Hz signals recorded from the neck are still unknown. Besides the direct dorsal column pathway, a postsynaptic dorsal column pathway has been identified in rodents and cats, which also conveys information about innocuous touch (*20*). The spinal projection neurons that constitute this pathway are located in the deep dorsal horn [Rexed laminae III-V; (*20*)]. Tactile processing in this gray matter region is modulated by input from supraspinal areas. Liu *et al.* (*19*) have shown that the pyramidal tract in rodents includes a projection from primary and secondary somatosensory cortex to interneurons in laminae III-V of the lumbar spinal cord, and that this pathway facilitates the detection of light touch. They proposed that this corticospinal circuit may mediate modulation of spinal sensory processing as a function of mental states (*19*). A projection from somatosensory cortex to the dorsal horn exists also in nonhuman primates (*26*). If a similar projection exists in humans, it could play an important role in the top-down modulation observed in our study.

The high-frequency signal was lateralized (Fig. 3A), as were components of the evoked response (fig. S9). Some degree of laterality may not be unexpected, given that the spinal cord preferentially processes tactile input from ipsilateral body parts (*27*). In addition, the noninvasively recorded high-frequency response may reflect a sum of several components, possibly including the lateralized, phase-locked plexus response. When attenuating phase-locked components by subtracting the trial-mean Fourier spectrum from single-trial Fourier spectra before computing power, we observed a pronounced reduction in power around the time of the peak of the high-frequency response (compare Fig. 3 and fig. S8). One reason for this could be that the highly phase-locked and highly lateralized plexus response contributes to the observed high-frequency signal, and, thus, to the laterality of its spatial pattern. In line with this idea, attenuating phase-locked responses reduced power primarily in ipsilateral channels (fig. S13). The modulation of high-frequency power by prior knowledge, which occurs slightly later than the high-frequency power peak, persisted after attenuating phase-locked components. A contribution of several components to the high-frequency response, some of which are phase locked, while others are phase jittered, could therefore explain why the observed modulation by prior knowledge did not coincide with the peak of the high-frequency response.

While muscle activity can generate high-frequency signals, a myogenic response of neck muscles cannot explain the observed modulation of high-frequency power by prior knowledge. This modulation occurs too early to reflect any motor commands from supraspinal regions in response to stimulation. The latency and duration of the observed high-frequency modulation are also shorter than the “somatomotor” responses of neck muscles to median nerve stimulation reported previously, which typically peak at ≥18 ms after median nerve stimulation, and whose duration is in the order of several tens of milliseconds (*28*). In contrast, we observe a modulation by prior knowledge that occurs earlier (13 to 16 ms) and lasts only a few milliseconds, consistent with a short burst of neuronal activity. Furthermore, power spectra of neck muscle activity typically drop above ~400 Hz (*29*), while the modulation we observe occurs in a circumscribed frequency window between 400 and 800 Hz. Last, because of their local reference, our invasive recordings are likely less susceptible to muscle activity than our noninvasive data but show a similar modulation of high-frequency signals by prior knowledge.

Somatosensory input to the spinal cord is gated by motor output (*30*–*32*). However, two control experiments ruled out the possibility that the observed high-frequency signal modulation was an indirect result of a change in muscle activity on the stimulated arm. Prestimulus muscle activity on the stimulated arm was similar between conditions (fig. S11). Furthermore, voluntary muscle contraction on the stimulated arm could not reproduce the pattern of short-latency, high-frequency signal modulation observed here (fig. S12). This is in agreement with previous epidural recordings from the human spinal cord, which have revealed that muscle activity does not alter short-latency, high-frequency signals below 1000 Hz (*10*).

Our invasive recordings revealed a shift in peak-power latency between conditions (Fig. 2A). It is not uncommon that cognitive factors shift the latency of early sensory responses (*33*). Previous studies have shown that attention can speed evoked responses to median nerve stimulation in primary somatosensory cortex (*34*) and the spinal cord (*35*). Given the absence of a significant latency shift in our noninvasive recordings, we cannot conclude with certainty that temporal predictability, too, speeds early responses. Should a systematic latency shift by a few milliseconds exist, it would not be a trivial result of condition differences in interstimulus intervals between the auditory cue and median nerve stimulation. The variation in interstimulus intervals in the variable-interval condition was several orders of magnitude higher than the observed high-frequency power latency shift, and included interstimulus intervals that were shorter or longer, than the interstimulus interval in the constant-interval condition (see also fig. S10). Future studies could employ paradigms with only few discrete interstimulus intervals, allowing for exact matching of interstimulus intervals despite differences in temporal expectation (*36*).

Our results do not exclude the possibility that prior knowledge modulates also non–phase-locked, low-frequency spinal cord responses to median nerve stimulation, in addition to high-frequency responses. We did not analyze low-frequency signals because the larger time windows required for spectral analysis of low-frequency signals, compared to high-frequency signals, prevent pinpointing any low-frequency signal modulations to the initial stages of somatosensory processing.

We demonstrate that short-latency 600-Hz signals from the human spinal cord are sensitive to prior knowledge. Given that 600-Hz signals in other parts of the nervous system are considered a signature of population spiking (*11*, *12*), the possibility to examine these signals noninvasively in humans raises exciting opportunities for future inquiries into the neurophysiology of the spinal cord in interaction with the brain, both in health and neurological disease.

## MATERIALS AND METHODS

### Subjects

We recruited 10 patients with neuropathic pain (age of 48 ± 10.3 years, mean ± SD; eight were female) who underwent implantation of an epidural lead with eight electrodes into the spinal canal (Boston Scientific; SC-2218 70 cm Linear ST Percutaneous Lead), alongside the dorsal cervical spinal cord. The lead was implanted to test the effectiveness of electric spinal cord stimulation for pain relief. Table S1 specifies the etiology and location of pain for each patient, as well as any central nervous system active medication. Two patients were excluded from the analyses because no evoked response could be obtained by median nerve stimulation. For one of these patients, this was likely due to the low maximum tolerable amplitude of median nerve stimulation (32.5 mA, 50 μs pulse width) and a pronounced motoric startle response to median nerve stimulation, which introduced noise into the recording. For the other patient, the reason for the absence of an evoked response was a mistake in the setup during recording. Of the included eight patients [age of 46.8 ± 11 years (mean ± SD)], seven were female.

In addition, we recruited healthy individuals via local participant databases and from students of Otto-von-Guericke University Magdeburg. Although we observed a large effect size in our invasive recordings, we expected noninvasive surface recordings to be noisier and show relatively smaller effects. We therefore chose a sample size of 30 healthy individuals, for which a power calculation revealed 90% power to detect effect sizes that were at least medium to large [Cohen’s *d* ≥ 0.6; using sampleSize_timefreq.m (*37*) in MATLAB/FieldTrip]. Healthy individuals were, on average, 26 years old (SD 3.2 years), and 12 were female.

All participants provided written informed consent. The study was approved by the ethics committee at the Medical Faculty at Otto-von-Guericke University Magdeburg (106/15), and conducted in accordance with the Declaration of Helsinki.

### Experimental task

Subjects were sitting comfortably in front of a computer monitor, wearing over-ear (patients) or in-ear headphones (healthy subjects). Noninvasive recordings were conducted in an electrically shielded, sound-attenuated chamber (Industrial Acoustics Company GmbH). Invasive recordings were conducted in a test cubicle at the hospital.

Our aim was to compare the spinal cord response to electric stimulation of the median nerve when the time of stimulation was highly predictable versus relatively unpredictable. Each trial started with the presentation of a sine wave tone via headphones (800 Hz, 100 ms duration), followed by electric stimulation of the median nerve, and a jittered intertrial interval (1500 to 3000 ms, uniform distribution). The auditory cue was presented to both ears simultaneously via soft polyurethane foam disposable ear buds in the noninvasive study, and via over-ear headphones in the invasive study. Audio volume was individually adjusted so that each participant could hear the beeping sound clearly, without experiencing discomfort. In alternating blocks of 100 trials each, the time interval between the tone and the median nerve stimulation was either constant (1100 ms; constant-interval condition), or varied randomly across trials (100 to 2100 ms, uniform distribution; variable-interval condition). Participants could thus form a precise prediction about the time of median nerve stimulation in any given trial of the constant-interval condition, but not the variable-interval condition. Three patients and 15 healthy subjects started with the constant-interval condition, while all remaining participants started with the variable-interval condition. We did not inform participants explicitly about the existence of two conditions or about differences in the consistency of the time interval between the cue and median nerve stimulation across blocks. Even without explicit instruction, humans form implicit temporal expectations (*38*, *39*).

To ensure that participants were attending to the median nerve stimulation, they had to count silently the number of targets in each block. A target was defined by a quick succession of two median nerve stimulations, separated by 80 ms. Each block contained between 5 and 10 targets (uniform distribution). At the end of each block, subjects verbally reported the number of targets in that block to the experimenter, who typed in the reported number. Subjects immediately saw feedback on a computer screen indicating how many targets there had been in the last block, and whether they had counted correctly. By asking for a single count at the end of each block, rather than a report after each individual stimulation, we avoided any confounding effects of muscle activity related to reporting during the block. However, this means we could not identify hits, misses, false alarms, and correct rejections on a single-trial basis.

To ensure that participants understood what a target was, they completed a first, short block of trials in the presence of the experimenter, and reported verbally after each median nerve stimulation whether it corresponded to a target, or not (no physiological data were recorded during this block). In this familiarization block, 50% of median nerve stimulations were targets. This allowed the experimenter to assess whether the participant had understood what a target was, in a reasonable amount of time. Because this high target frequency during familiarization induced a response bias in the first block of the main experiment in our invasive study, we informed participants in our noninvasive study before the main experiment that the frequency of targets would be lower than during familiarization.

Patients completed 4 (one patient), 6 (five patients), or 10 blocks (two patients) of the main experiment in a single session. Of the 30 healthy subjects, 29 completed between 28 and 32 blocks of the main experiment, distributed across 2 days of testing (consecutive days in 21 subjects, and separated by 3 to 9 days in the remaining subjects). One healthy subject completed only a single day of testing (13 blocks).

### Median nerve stimulation

We electrically stimulated the median nerve via surface electrodes at the wrist (Ambu Neuroline 700) using a DS7 stimulator for all patients, and a DS5 stimulator for all healthy subjects (Digitimer Ltd.). None of our participants experienced the electric stimulation as painful. In patients, we stimulated the body side that was unaffected by neuropathic pain (left in four patients). In all healthy subjects, we stimulated the left median nerve. DS7 and DS5 delivered a constant current square wave pulse. For patients, whose data were collected first, we chose the shortest possible pulse width (50 μs). Given that we observed a similar high-frequency signal also with longer pulse widths (*9*), we opted for a standard pulse width of 200 μs for healthy subjects. In all cases, the proximal electrode for median nerve stimulation was positive. Stimulation amplitude was individually set at a level that elicited a reliable, visible thumb twitch [mean of 57.25 mA and range of 35 to 80 mA (at 50 μs pulse width), in patients; mean of 6.84 mA and range of 4.5 to 9.3 mA (at 200 μs pulse width), in healthy subjects]. For four healthy subjects, the amplitude of median nerve stimulation had to be temporarily adjusted during the experiment (for one block in three subjects, and for five consecutive blocks in one subject) because subjects reported a decrease of perceived stimulation intensity, possibly due to a change in wrist position. For these subjects, we ensured that our analysis matched the numbers of blocks with identical median nerve stimulation amplitude between the two conditions. For the three subjects for whom stimulation amplitude was adjusted for a single block, that block was excluded from the analysis. For the subject for whom stimulation amplitude was adjusted for five consecutive blocks, the first of these blocks was excluded from the analysis, so that the adjusted stimulation amplitude affected two included blocks of each condition (given that conditions alternated by block).

### Recording of spinal cord potentials

We conducted invasive recordings on the second or third postoperative day, when the lead was still externalized as part of the standard medical procedure. In each patient, we recorded from eight epidural contacts (3 mm length and 1 mm spacing) placed alongside the dorsal cervical spinal cord, using a BrainAmp DC amplifier (Brain Products GmbH) at a resolution of 0.1 μV. As a reference, we chose the fourth contact (counted from the most caudal contact), thus recording seven channels. Choosing the fourth electrode, as one of the middle electrodes on the lead, allowed us to keep the largest distance between any of the eight electrodes and the reference to a minimum, thereby emphasizing nearby sources across the entire array of electrodes. An extra wire twisted around the wire for the sixth contact (counted from the most caudal contact) served as ground. The sampling rate was 2500 Hz, with an anti-aliasing filter set to 1000 Hz. To avoid saturation due to low-frequency drifts, the hardware high-pass filter was set to 0.016 Hz. Spinal cord stimulation was turned off during recording. Both data acquisition and analysis of the invasive study were finished before the start of the noninvasive study.

For noninvasive recordings in healthy subjects, we attached 24 EEG ring electrodes (Easycap, outer diameter 12 mm) via electrode holders and double adhesive electrode washers to the skin around the neck, extending an established cervical electrode montage (*6*, *9*). Our motivation to include a larger number of electrodes than in our previous study (*9*) was to exploit redundancy in the recording via spatial filtering (*16*). Electrodes were arranged in three ring formations around the neck (Fig. 1B). For the middle ring, one electrode was placed above the spinous process of the sixth cervical vertebra and one onto the thyroid cartilage. Between these two electrodes, the remaining six electrodes for the middle ring were evenly spaced around the neck. For the caudal and cranial rings, electrodes were placed approximately 2 cm below and 2 cm above the corresponding electrodes of the middle ring, respectively. In addition, we recorded the ECG via an electrode placed on the left lateral epicondyle. Reference and ground electrodes were placed on the right and left acromion, respectively. We filled the void between the electrodes and the skin with conductive abrasive gel. Impedances were kept below 5 kilohms. We used a BrainAmp DC amplifier (Brain Products GmbH) at a resolution of 0.1 μV, with a sampling rate of 5000 Hz, an anti-aliasing filter set to 1000 Hz, and a hardware high-pass filter set to 0.016 Hz.

### Data analysis

We used MATLAB (version 9.6.0., R2019a) for analysis of physiological data, which used the FieldTrip Toolbox (*40*), Noise Toolbox (*16*, *41*), and custom-written scripts. Behavioral data were analyzed in MATLAB and JASP (*42*).

Given that invasive and noninvasive recordings differed in recording sites, number of channels, and consistency of electrode placement across subjects, the first analysis step differed between invasive and noninvasive recordings. In both cases, we first derived a virtual channel that captured the spinal cord response to median nerve stimulation, however, with a different purpose, and different approach (as described in the “Virtual channel” section). All subsequent preprocessing steps of virtual channel data were identical for invasive and noninvasive data (as described in the “Preprocessing and time-frequency transformation” section).

#### Virtual channel

To obtain a virtual channel that captured the spinal cord response to median nerve stimulation, we used an established spatial filtering technique called denoising separation of sources (DSS) (*16*), both for invasive and noninvasive data. DSS provides a spatial filter, i.e., weights for a linear combination of the recorded channels, by jointly diagonalizing two covariance matrices. One is the covariance matrix of the data after “bias filtering” to emphasize a given signal of interest (C1) and the other is the covariance matrix of the original, “unfiltered” data (C0). Depending on the signal of interest, a bias filter does not necessarily correspond to a spectral (convolutional) filter, but may instead correspond to the selection of a specific time window of interest, or to averaging across trials (*16*). Bias filters were not set up to emphasize differences between conditions, but instead based on all trials across both conditions. Condition differences were investigated only after spatial filtering, in the obtained virtual channel.

The purpose of spatial filtering, and the filtering approach, differed between invasive and noninvasive data. For patients, the exact placement of epidural electrodes along the craniocaudal axis of the cervical spinal canal was determined on the basis of clinical considerations and varied across individuals (fig. S1A). We therefore expected interindividual variation in the distribution of the spinal cord response to median nerve stimulation across the seven recorded channels. Thus, the purpose of spatial filtering in our invasive study was to obtain a virtual channel that captured the spinal cord response to median nerve stimulation in each patient and then perform group-level analyses for this channel. We therefore used spatial filtering at a single-subject level, with the goal to capture the segmental response to median nerve stimulation in the spinal cord.

We expected the segmental response to consist of an evoked (phase-locked) response and a high-frequency response. Given that we had to construct a separate virtual channel for each patient (because of interindividual differences in electrode placement), we chose a bias filter that emphasized the signal with the higher expected signal-to-noise ratio. Because averaging across trials increases the signal-to-noise ratio, this was the evoked response. Our bias filter was therefore set up to emphasize phase-locked responses (see fig. S14 for a confirmation that the alternative approach, i.e., to emphasize high-frequency signals directly, was less successful). To construct a bias filter that emphasized phase-locked responses, we averaged across trials before computing C1. Specifically, we computed C0 from data epoched between 5 and 20 ms after median nerve stimulation and C1 from the average across these epochs. The distribution of power ratios of all obtained components indicated that the first component captured phase-locked responses in each patient, as evident in a sharp decline in power ratios from the first component to the second component (fig. S1B). Furthermore, in most patients, a resampling test provided evidence that the first component did not merely capture noise (shading in fig. S1B). Our time-frequency analysis confirmed that the first component also captured the high-frequency response (Fig. 2), consistent with an overlapping, i.e., segmental, source for both signals. Figure S1A displays each patient’s spatial pattern (i.e., inverse of the filter coefficients) for this component. All further preprocessing steps were applied to component-level (virtual-channel) data and identical to the preprocessing of component-level data obtained from the noninvasive recordings (“Preprocessing and time-frequency transformation” section).

Extraction of a virtual channel from noninvasive data served a different purpose. Spatial filtering can enhance the signal-to-noise ratio (*16*) and is recommended for efficient analysis of high-frequency signals in noninvasive recordings (*43*), including recordings from the human spinal cord (*9*). On the basis of our invasive recordings, we expected a difference between constant-interval and variable-interval conditions in short-latency, high-frequency power between 400 and 800 Hz. We therefore tailored spatial filters to capture the expected stimulation-induced increase in power in this time-frequency window. We computed C0 and C1 based on data that were spectrally filtered between 400 and 800 Hz and contrasted two time intervals: a short-latency time window, for which we expected a high-frequency response to median nerve stimulation (8 to 16 ms, for C1), and a more extended, “full” time window (8 to 200 ms, for C0) (*9*, *43*). This approach allowed us to avoid any spectral (convolutional) filtering of the data that were eventually analyzed. Instead, spectral filtering was only used in the construction of the spatial filter, while the data subsequently projected through this filter, and shown throughout the manuscript, are not spectrally filtered, and therefore uncontaminated by the artifacts that convolutional filters can introduce (*44*).

Specifically, we first removed the ECG artifact from the raw, continuous data using template subtraction (*9*), then interpolated the electric stimulation artifact, and created a bandpass filtered (400 to 800 Hz, fourth-order Butterworth filter) copy of the continuous data. Both the unfiltered and the spectrally filtered data were epoched between 0 and 200 ms after median nerve stimulation. Bad channels were identified on the basis of notes during recording, and via a threshold to the robust *z* score (*45*) of the unfiltered data (threshold of 3), and interpolated (average of their neighbors). Trials were excluded from the construction of the spatial filter if the amplitude variance over time (in the channel with the maximum amplitude variance over time) exceeded a threshold of 1000 μV. This coarse trial rejection criteria was applied to avoid biasing of the spatial filter by obvious outlier trials. For the subsequent analysis at the component-level, a different trial rejection criterion was used instead, which was specifically tailored to our interest in high-frequency signals (see the “Preprocessing and time-frequency transformation” section).

The consistent montage used for noninvasive recordings across individuals allowed for construction of a spatial filter at the group level. Trial-wise data were concatenated across subjects and (trial-wise) *z* transformed (*9*). We then computed C0 and C1 in the abovementioned time windows, and derived a spatial filter via DSS. On the basis of the distribution of power ratios of all obtained components (Fig. 3A, right) we selected only the first component, i.e., the component expected to reflect the strongest increase in 400 to 800 Hz power in our time window of interest (*9*). Figure 3A, right, also shows the results of a resampling test with 1000 iterations. For each iteration, we recomputed C1 after randomly selecting the 8-ms time window for which C1 was computed from an overall time window between 16 and 200 ms after median nerve stimulation, for each trial separately. This test provides an estimation of power ratios expected under the null hypothesis that there is purely noise in the time window between 8 and 16 ms after median nerve stimulation. The gray shading in Fig. 3A, right, represents the 5 to 95% percentile range of power ratios under this null hypothesis.

#### Preprocessing and time-frequency transformation

For invasive and noninvasive recordings, virtual channel data were first epoched between −1500 ms and +1500 ms relative to median nerve stimulation. We included epochs that contained a target (two consecutive median nerve stimulations), given that the second stimulation in these trials occurred 80 ms after the first, and thus did not affect our time window of interest (8 to 16 ms after the first median nerve stimulation). The second stimulation in a target was not included. We interpolated the electric stimulation artifact between 0 and 4 ms after stimulation (pchip.m), and attenuated line noise at 50 Hz, and harmonics up to 800 Hz, using spectrum interpolation (*46*) in FieldTrip (setting “dftreplace” to “neighbour_fft,” “dftbandwidth” to 3 Hz, and “dftneighbourwidth” to 10 Hz). Data were then time-frequency transformed trial-wise at all sampling points between −550 ms and +50 ms relative to median nerve stimulation, and between 200 and 1200 Hz, in steps of 50 Hz, using FieldTrip’s multitaper method (mtmconvol) in combination with a frequency-dependent Hanning taper (5 cycles). In separate analyses, we computed power after subtracting the trial-mean Fourier spectrum from single-trial Fourier spectra, thereby attenuating phase-locked responses (Fig. 3 and figs. S4 to S7 and S10), or we first averaged time-domain data across trials and then computed the time-frequency transformation to examine to what extent condition differences were reflected in phase-locked responses (fig. S3). Data were then single-trial, full-epoch length baseline corrected (*47*) [where a “full epoch” corresponds to the time window used for subsequent analysis, i.e., −500 to +20 ms, excluding ±10 ms around the (interpolated) median nerve stimulation]. Trials were excluded from further analysis if they exceeded a threshold of at least one of two robust *z* scores (computed across conditions). Robust *z* scores were computed for the maximum power in each trial (in decibels, taking the maximum across frequency and time), and for the maximum variance in power over time (in decibels, where the maximum is computed across frequencies). The threshold was set to 5, resulting in a rejection of <1% of all trials on average across individual (invasive data, 0.07% ± 0.1%; and noninvasive data, 0.75% ± 0.1%; mean ± SD). For the invasive study, the numbers of trials available per condition (range across patients, 199 to 500) did not differ by more than a single trial between conditions in any of the patients. For the noninvasive study, the numbers of trials available per condition ranged from 593 to 1664 [means ± SD, 1545.5 ± 174.48 trials (constant-interval condition) versus 1550.1 ± 183.63 trials (variable-interval condition)].

Included trials were separated by condition, and power was averaged within conditions, and expressed in decibels relative to baseline. As a baseline, we chose a time window between −500 and −10 ms relative to median nerve stimulation. In a control analysis, we selected a baseline before onset of the auditory cue (between −500 and −10 ms relative to the onset of the auditory cue; fig. S6).

### Statistics

We compared spectral power, and the amplitude of the evoked response, between conditions using FieldTrip’s cluster-based permutation test, which corrects for multiple comparisons across time and frequency bins (*48*) [given that tests were run at the component-level, there was only a single (virtual) channel]. All tests were two sided. For the invasive data, we computed the cluster-level statistic for all 256 (2^8^) possible permutations, and contrasted spectral power in our a priori (*9*) time window and frequency window of interest (8 to 16 ms and 200 to 1000 Hz). This specific frequency window was motivated by our previous study (*9*), with the only difference that we chose an upper limit of 1000 Hz, rather than 1200 Hz, given the cutoff frequency of the anti-aliasing filter of our amplifier (1000 Hz). Having established in our invasive recordings a condition effect between 13 and 16 ms, peaking at around 600 Hz, statistical analysis of the noninvasive data focused on a time window between 13 and 16 ms, and a frequency window between 400 and 800 Hz. For the noninvasive data, we computed 500 random permutations. In a control analysis, we compared data from all trials in the constant-interval condition with data from the variable-interval trials in which the time interval between the auditory cue and median nerve stimulation fell within a range of 1100 ± 200 ms (fig. S10). A range of interstimulus intervals, rather than an exact matching to the constant-interval condition, was necessary because the number of variable-interval trials with an interstimulus interval of exactly 1100 ms was very low. The specific range of ±200 ms was chosen because it yielded more than 100 trials per subject (315.93 trials on average, minimum 115).

We used a dependent-samples *t* test in JASP to compare the proportion of blocks for which participants counted the number of targets correctly. Given that the high frequency of targets in the initial familiarization block induced a response bias in the first block of the main experiment (patients miscounted by 7, on average, in that block), we excluded that block from the behavioral analysis of our invasive study (statistical results were qualitatively unchanged when including that block). In one patient, the response bias persisted throughout the experiment (P006). Excluding P006 from behavioral and physiological analyses did not result in any qualitative change to statistical results. For the noninvasive study, we informed all participants before the start of the main experiment that targets would now be much rarer. This effectively prevented any carryover of response bias from the familiarization block to the first block of the main experiment (median counting error in the first block: 0). Therefore, no block was excluded from the behavioral analysis of the noninvasive study.
