# Supplementary material for: Prior knowledge changes initial sensory processing in the human spinal cord
Source: Sci Adv. 2025 Jan 15;11(3):eadl5602. doi: 10.1126/sciadv.adl5602 (PMC11734707; doi:10.1126/sciadv.adl5602)
Supplement: Supplementary file 1 — Figs. S1 to S14 Table S1 [file sciadv.adl5602_sm.pdf]

Science Advances
AAAS

Supplementary Materials for

Prior knowledge changes initial sensory processing in the human spinal cord

Max-Philipp Stenner *et al.*

Corresponding author: Max-Philipp Stenner, max-philipp.stenner@lin-magdeburg.de

Sci. Adv. **11**, eadl5602 (2025)
DOI: 10.1126/sciadv.adl5602

This PDF file includes:

Figs. S1 to S14
Table S1

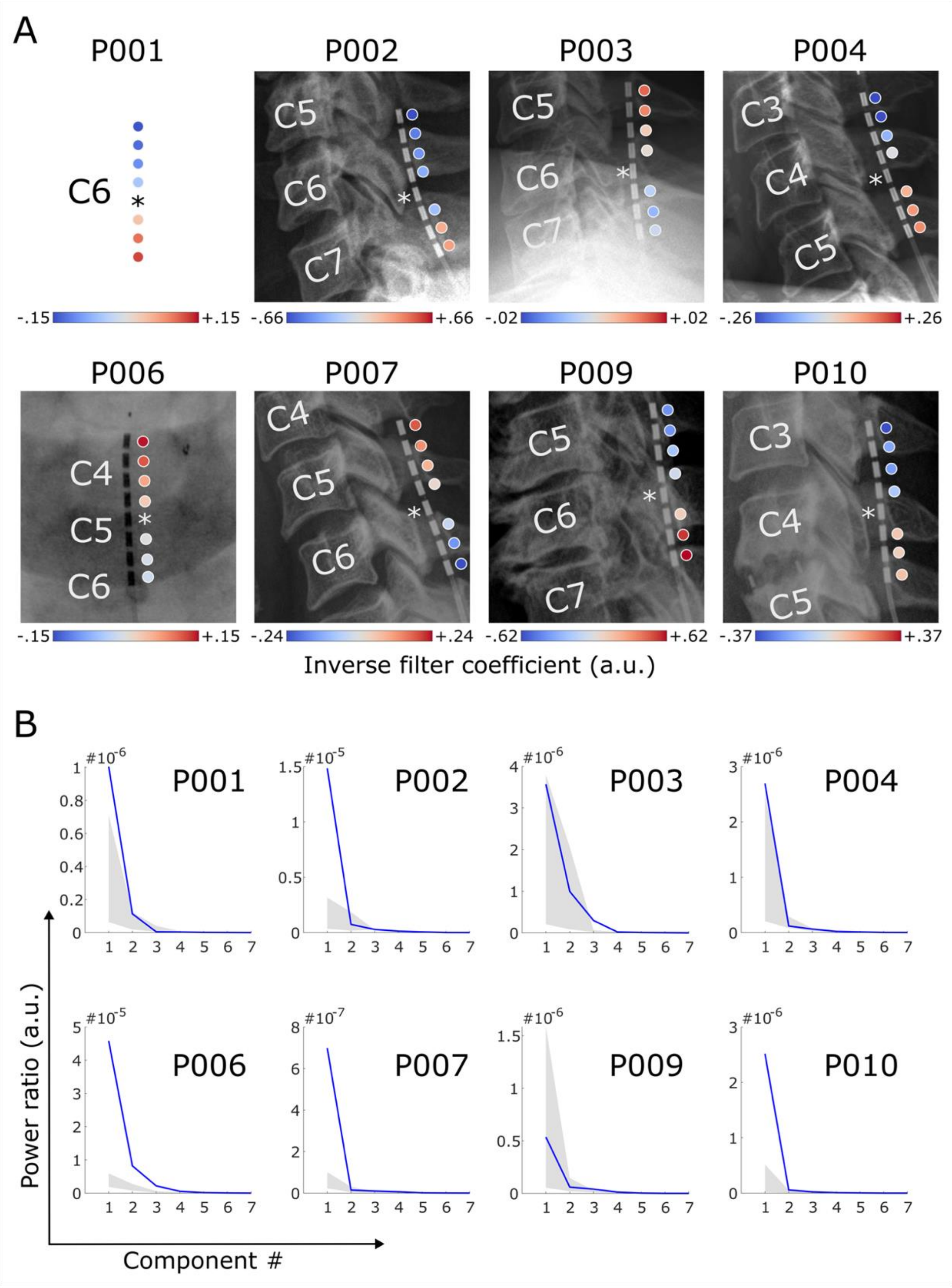

Figure S1.

Electrode locations, spatial patterns, and power ratios for the spatial filter derived for each patient. Each panel in **A** shows a post-operative X-ray of the cervical spinal cord for each patient, together with the spatial pattern (the inverse of the filter coefficients) of the first component derived from spatial filtering. The star indicates the electrode used as a reference. Color codes for the inverse of filter coefficients. For patient P001, no X-ray was available. Each panel in **B** shows the power ratio for the seven components for each patient (blue), together with results of a resampling test (grey shading). For this test, we re-computed power

ratios after randomly selecting the 15 ms-duration time window from which covariance matrix C1 was computed from a time window between 20 and 200 ms after median nerve stimulation, for each trial separately. This test provides an estimation of power ratios expected under the null hypothesis that there is purely noise in the time window between 5 and 20 ms after median nerve stimulation. The shading in B represents the 5% to 95% percentile range of power-ratios under this null hypothesis. In two patients, the observed power ratio for the first component did not exceed the 95% percentile (P003 and P009), however, in each of these patients, that component nevertheless showed a clear evoked response.

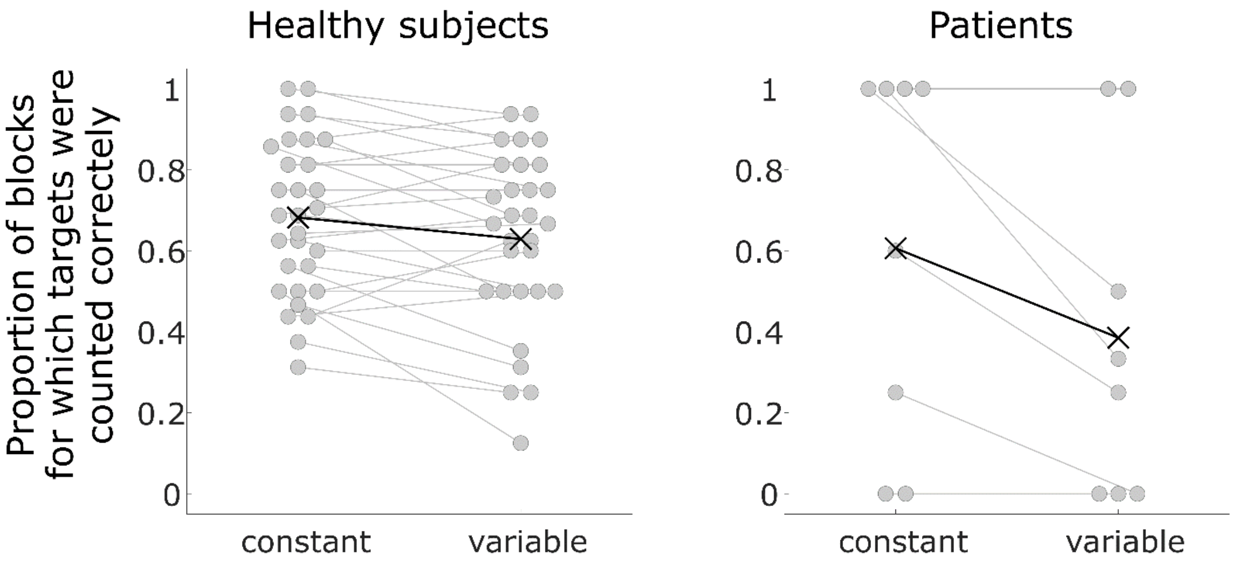

Figure S2.

Proportion of blocks for which healthy subjects (left) and patients (right) counted targets correctly, in the constant-interval condition and variable-interval condition. Each individual is represented by a grey dot. The cross represents the mean across subjects. Importantly, even in blocks in which they did not count correctly, all healthy participants miscounted by only 1 to 2, and all patients, except for one, miscounted by only 1 to 2.5 (range across subjects of medians across blocks). Only a single patient miscounted by more (by around 50), likely because of a shifted response criterion induced by the high frequency of targets (50%) in a training block at the beginning of the experiment, which served to familiarise participants with targets. The difference in the proportion of blocks in which targets were counted correctly persisted when excluding this patient (P006; $t(6)=2.48$, $p=.048$, $d=0.94$; 69.29 ± 38.95% (constant-interval condition) vs. 44.05 ± 39.01% (variable-interval condition; mean ± standard deviation)).

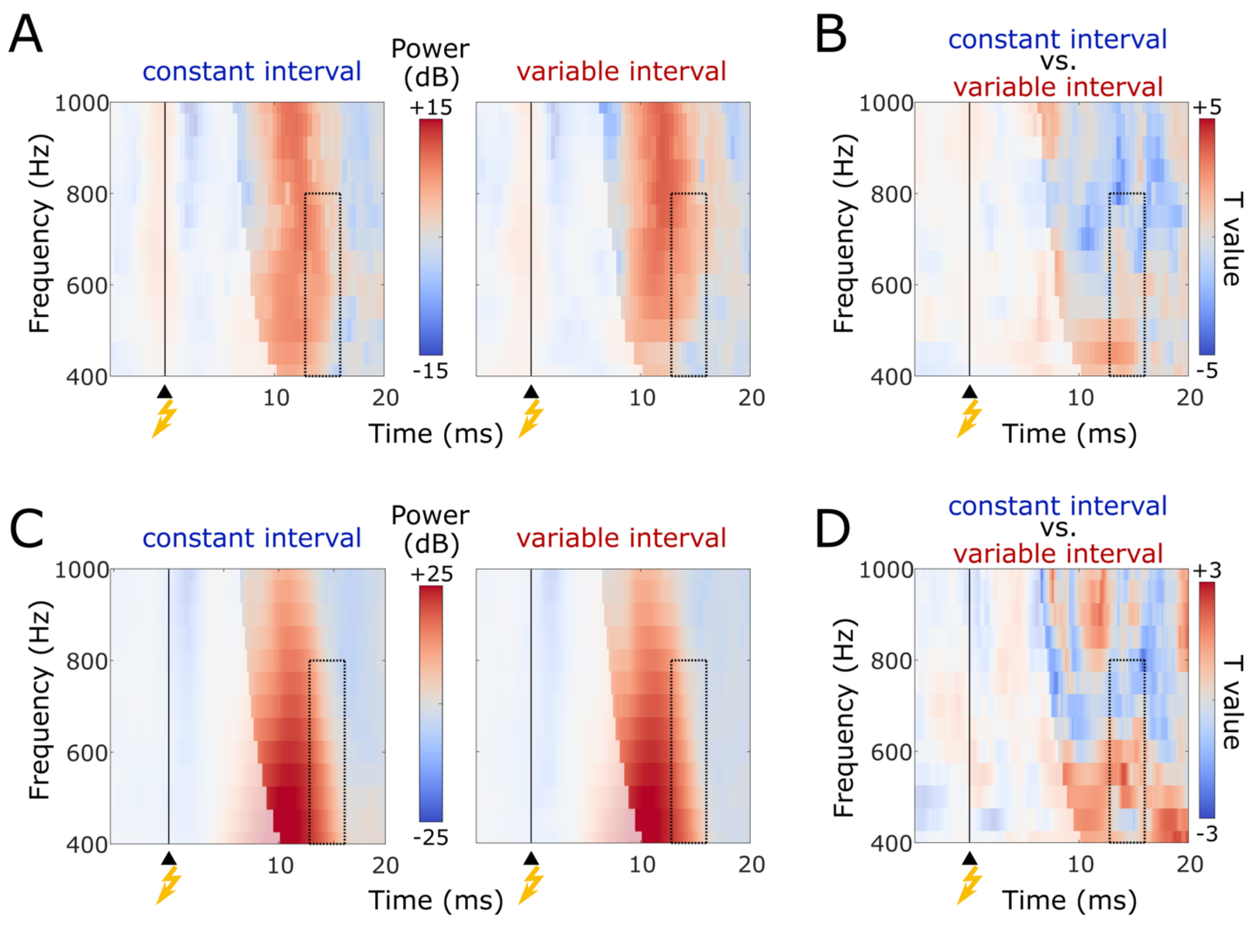

Figure S3.

Time- and frequency-resolved power, computed *after* averaging across trials, in the constant- and variable-interval conditions for invasive (A, B) and non-invasive (C, D) recordings. Panels A and C show grand means, and panels B and D show T values at the group level. No significant differences between conditions were found for data recorded invasively (A, B, p>.8) and non-invasively (C, D, p>.6). The contours in all panels represent the spectro-temporal extent of the time-frequency window for which we observed a significant difference between conditions when computing power for individual trials (Figures 2 and 3 in the manuscript), i.e., when time-frequency transformation preceded averaging across trials (frequency window between 400 Hz and 800 Hz, and time window between 13 and 16 ms). Time-frequency bins possibly affected by the stimulation artefact, given the frequency-dependent taper (5 cycles), are semi-opaque. In all panels, the yellow flash on the x-axis indicates the time of median nerve stimulation.

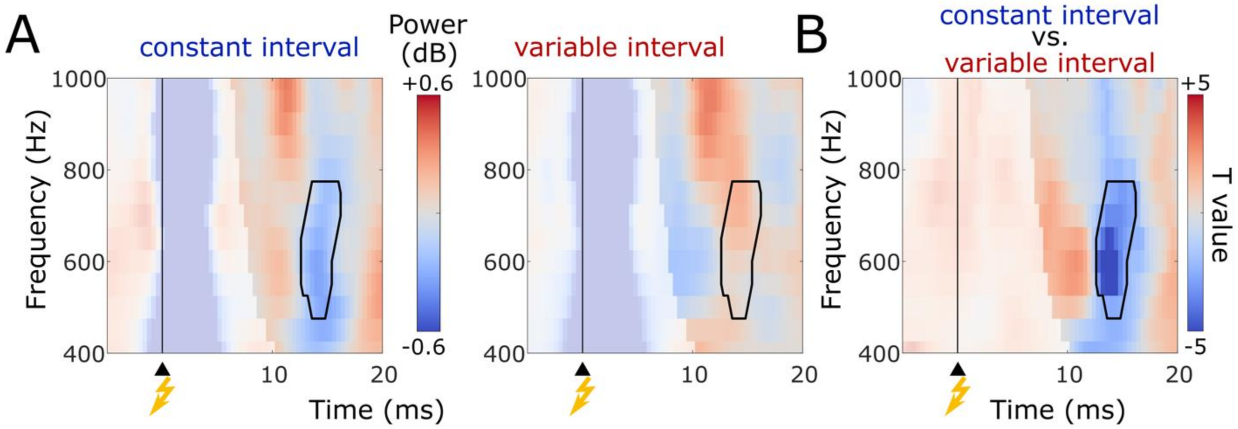

Figure S4.

Time- and frequency-resolved power in the constant- and variable-interval conditions, computed after attenuating phase-locked responses in the invasive recordings. Panel **A** shows grand means, and panel **B** shows T values at the group level. The contour represents the spectro-temporal extent of the cluster observed in the cluster-based permutation test. This test showed that power in a frequency window between 200 Hz and 1000 Hz, and in a time window between 8 and 16 ms, was significantly lower in the constant-interval condition, compared to the variable-interval condition (p=.023, cluster-based permutation test; d=2.09). Time-frequency bins possibly affected by the stimulation artefact, given the frequency-dependent taper (5 cycles), are semi-opaque. In all panels, the yellow flash on the x-axis indicates the time of median nerve stimulation.

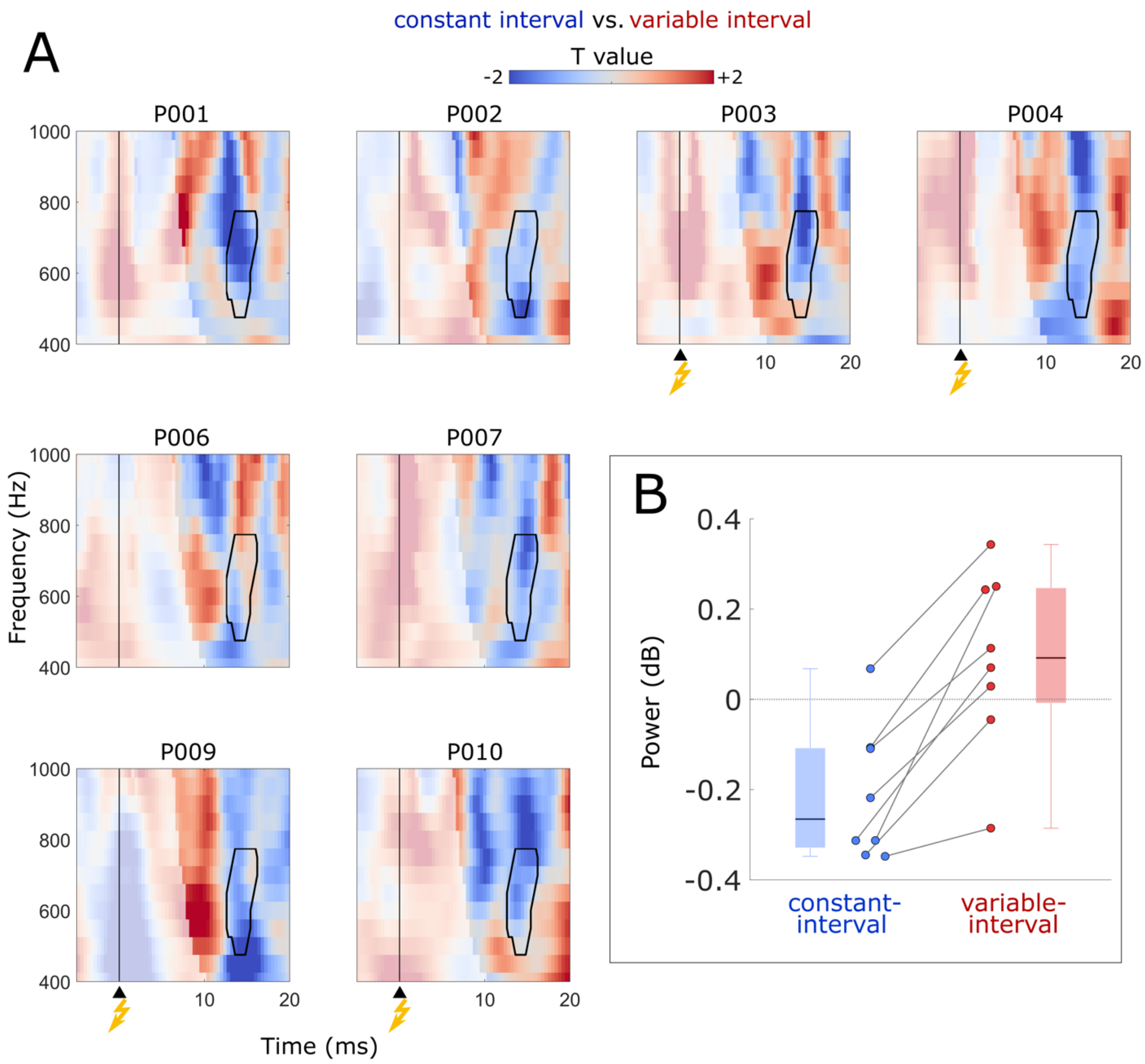

Figure S5.

Within-subject differences in time- and frequency-resolved power between the constant- and variable-interval conditions, computed after attenuating phase-locked responses in the invasive recordings. All panels in **A** show T values of an independent-samples T test at an individual-subject level, comparing trials across conditions. Each panel represents one patient. Time-frequency bins possibly affected by the stimulation artefact, given the frequency-dependent taper (5 cycles), are semi-opaque. The contour represents the spectro-temporal extent of the cluster observed in the cluster-based permutation test at the group-level (same as Figure S4). In all panels, the yellow flash on the x-axis indicates the time of median nerve stimulation. Panel **B** shows mean power across this cluster for each individual and condition (two dots connected with a line represent one individual).

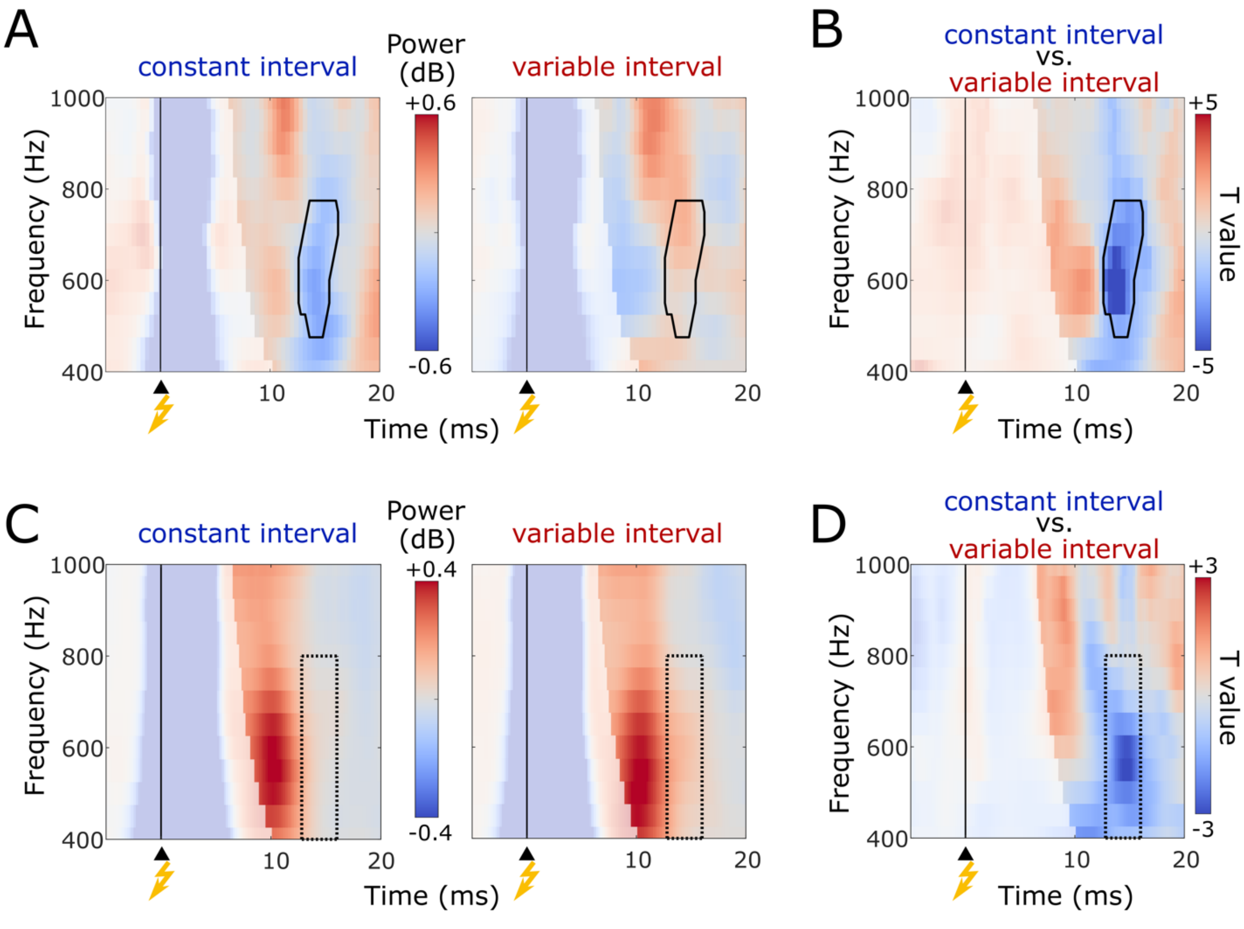

Figure S6.

Time- and frequency-resolved power, relative to a pre-cue baseline (-500 to -10 ms), in the constant- and variable-interval conditions for invasive (A, B) and non-invasive (C, D) recordings. All panels show data after attenuating phase-locked responses. Panels A and C show grand means, and panels B and D show T values at the group level. The contour in panels A and B represents the spectro-temporal extent of the cluster observed in the cluster-based permutation test. This test showed that power in a frequency window between 200 Hz and 1000 Hz, and in a time window between 8 and 16 ms, was significantly lower in the constant-interval condition, compared to the variable-interval condition (p=.023, cluster-based permutation test; d=2.1). The contour in panels C and D represents the time-frequency window used for the cluster-based permutation test for non-invasive data (400 Hz to 800 Hz, and 13 to 16 ms). This test revealed that power was significantly lower in the constant-interval condition, compared to the variable-interval condition (p=.009; d=.658). Time-frequency bins possibly affected by the stimulation artefact, given the frequency-dependent taper (5 cycles), are semi-opaque. In all panels, the yellow flash on the x-axis indicates the time of median nerve stimulation.

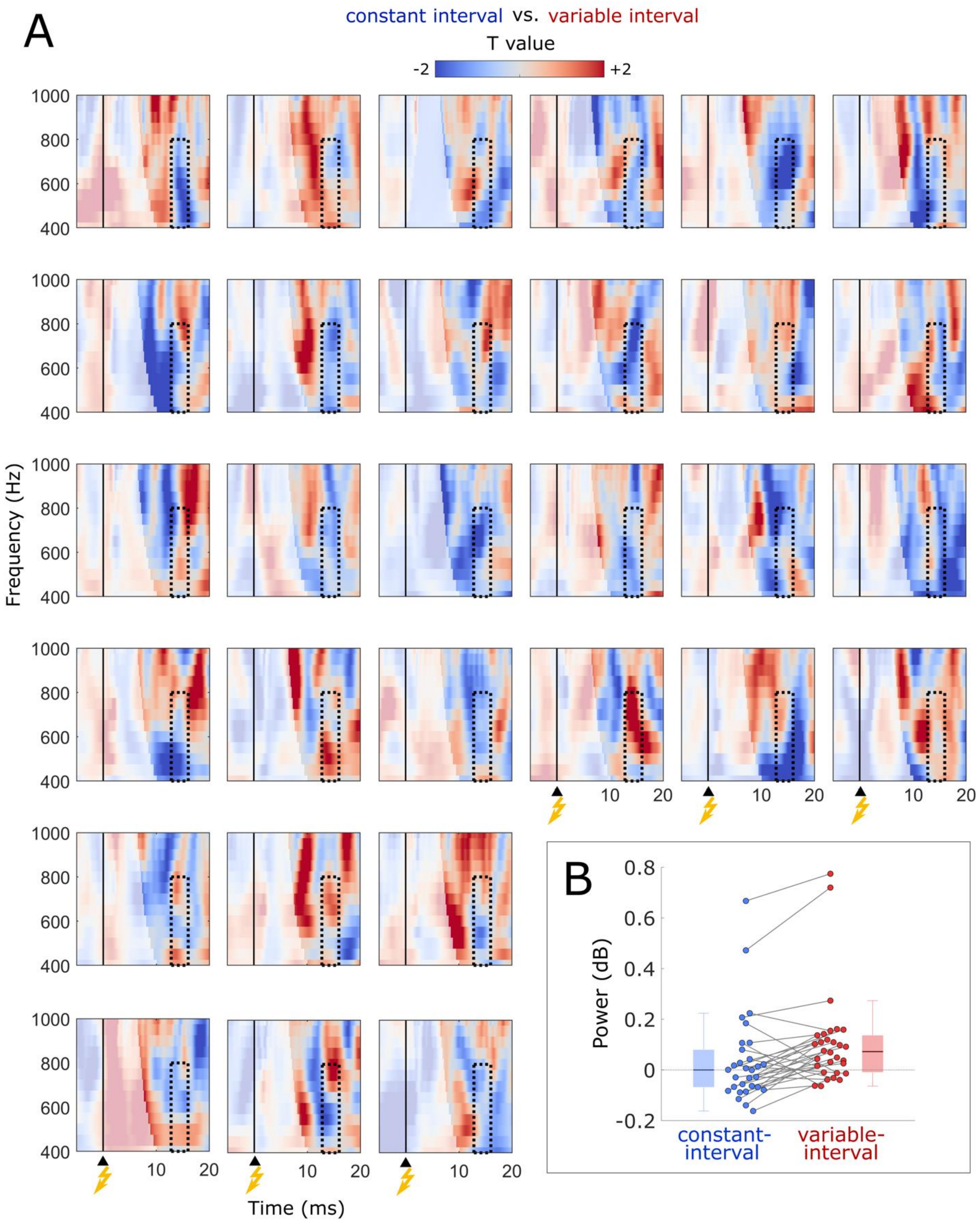

Figure S7.

Within-subject differences in time- and frequency-resolved power between the constant- and variable-interval conditions, computed after attenuating phase-locked responses in the non-invasive recordings. All panels in **A** show T values of an independent-samples T test at an individual-subject level, comparing trials across conditions. Each panel represents one participant. Time-frequency bins possibly affected by the stimulation artefact,

given the frequency-dependent taper (5 cycles), are semi-opaque. The contour represents the time-frequency window of interest for the cluster-based permutation test at the group-level (400 to 800 Hz, 13 to 16 ms). In all panels, the yellow flash on the x-axis indicates the time of median nerve stimulation. Panel **B** shows mean power across this cluster for each individual and condition (two dots connected with a line represent one individual).

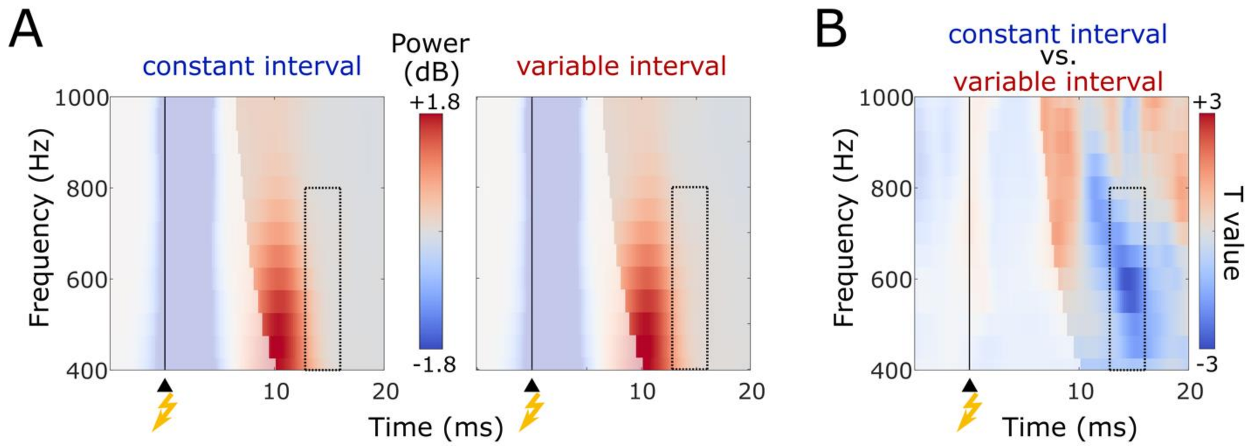

Figure S8.

Time- and frequency-resolved power in the constant- and variable-interval conditions, computed without attenuating phase-locked responses in the non-invasive recordings. Panel **A** shows grand means, and panel **B** shows T values at the group level. The contour represents the time-frequency window used for the cluster-based permutation test for non-invasive data (400 Hz to 800 Hz, and 13 to 16 ms). This test revealed that power was significantly lower in the constant-interval condition, compared to the variable-interval condition ($p=.018$; $d=.667$). Time-frequency bins possibly affected by the stimulation artefact, given the frequency-dependent taper (5 cycles), are semi-opaque. In all panels, the yellow flash on the x-axis indicates the time of median nerve stimulation.

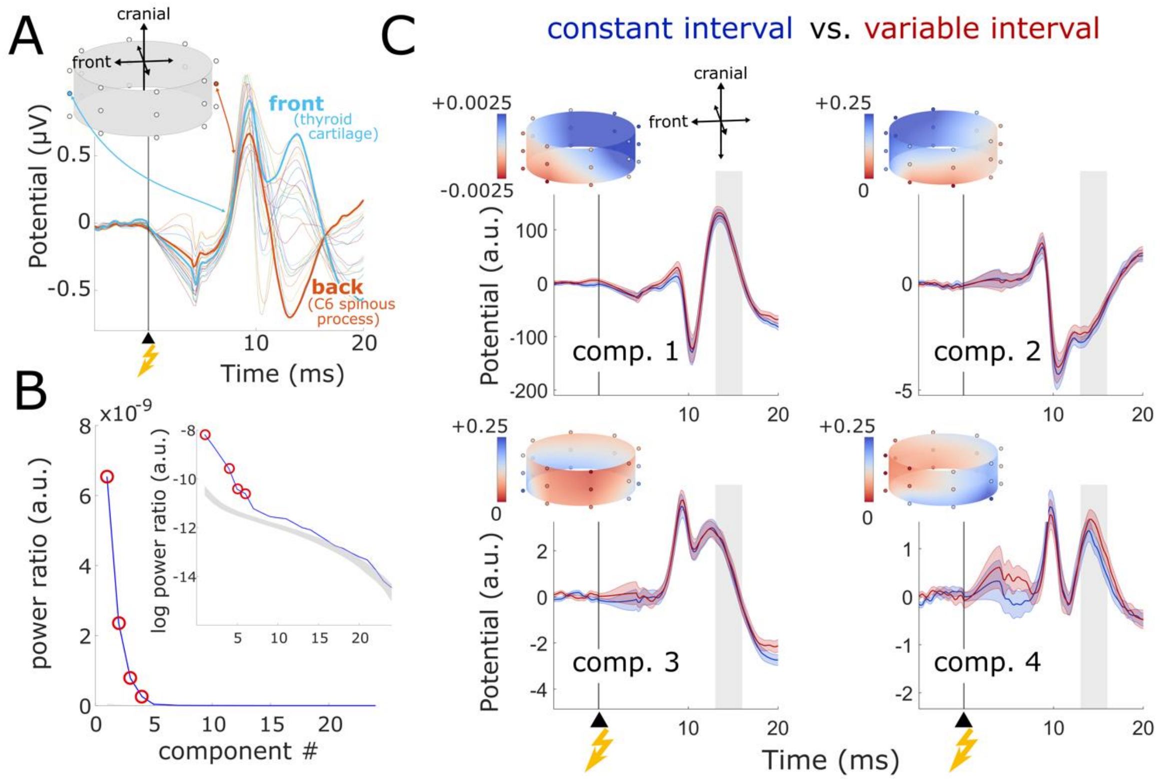

Figure S9.

Evoked response in non-invasive recordings at the sensor level (A), and after applying a spatial filter that emphasizes phase-locked responses (B, C). Of the 24 channels shown in A, two channels at the back and front of the neck are highlighted to illustrate the polarity reversal that is characteristic for the N13/P13 component, a typical spinal cord response of presumed segmental origin. B, power ratios for a spatial filter that emphasizes phase-locked components by jointly diagonalizing two covariance matrices, one computed from single-trial data between 8 and 200 ms after median nerve stimulation (C0), and one computed from trial-averaged data between 8 and 16 ms (C1). The grey shading represents results of a resampling test, for which we re-computed power ratios after randomly selecting the 8 ms-duration time window from which covariance matrix C1 was computed from a time window between 16 and 200 ms after median nerve stimulation, for each trial separately. This test provides an estimation of power ratios expected under the null hypothesis that there was purely noise in the time window between 8 and 16 ms after median nerve stimulation. The shading in B represents the 5% to 95% percentile range of power ratios under this null hypothesis. The red circles highlight the power ratios of the first four components, whose time courses are displayed in panel C, for each condition separately, together with their spatial patterns. None of the components showed a condition difference between 13 to 16 ms (grey shading), nor in a broader time window (8 to 16 ms; cluster-based permutation test, all $p>.3$). Bayesian paired-samples t-tests (or, in cases where the assumption of normality was violated, Bayesian Wilcoxon signed-rank tests) revealed moderate evidence in favour of the null hypothesis of no condition difference (all $BF_{01}>3$). In panels A and C, the yellow flash on the x-axis indicates the time of median nerve stimulation.

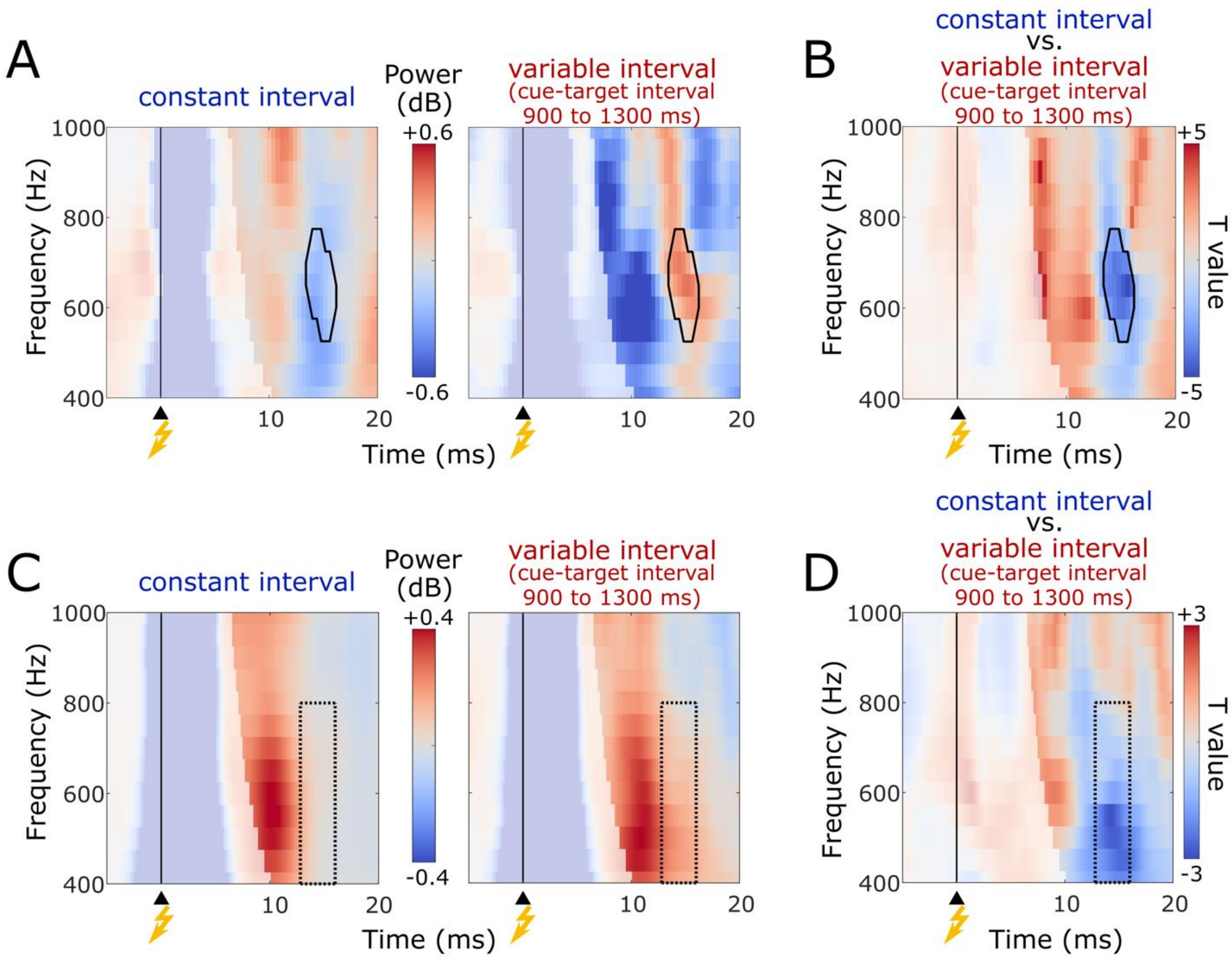

Figure S10.

Time- and frequency-resolved power for invasive (A, B) and non-invasive (C, D) recordings, comparing constant-interval trials to those variable-interval trials for which the time interval between the auditory cue and median nerve stimulation fell within a range of 1100 ± 200 ms. Panels A and C show grand means, and panels B and D show T values at the group level. The contour in panels A and B represents the spectro-temporal extent of a cluster observed in the cluster-based permutation test. This test showed that power in a frequency window between 200 Hz and 1000 Hz, and in a time window between 8 and 16 ms, tended to be lower in the constant-interval condition, compared to the variable-interval condition (p=.094, cluster-based permutation test; d=1.61). Excluding the single patient for whom less than 50 variable-interval trials were available with an inter-stimulus interval of 1100 ± 200 ms (P006), we observed a significant condition difference (p=.016, cluster-based permutation test; d=1.97). The contour in panels C and D represents the time-frequency window used for the cluster-based permutation test for non-invasive data (400 Hz to 800 Hz, and 13 to 16 ms). This test revealed that power was significantly lower in the constant-interval condition, compared to trials in the variable-interval condition with an inter-stimulus interval in the range of 1100 ± 200 ms (p=.007; d=.628). Time-frequency bins possibly affected by the stimulation artefact, given the frequency-dependent taper (5 cycles), are semi-opaque. In all panels, the yellow flash on the x-axis indicates the time of median nerve stimulation.

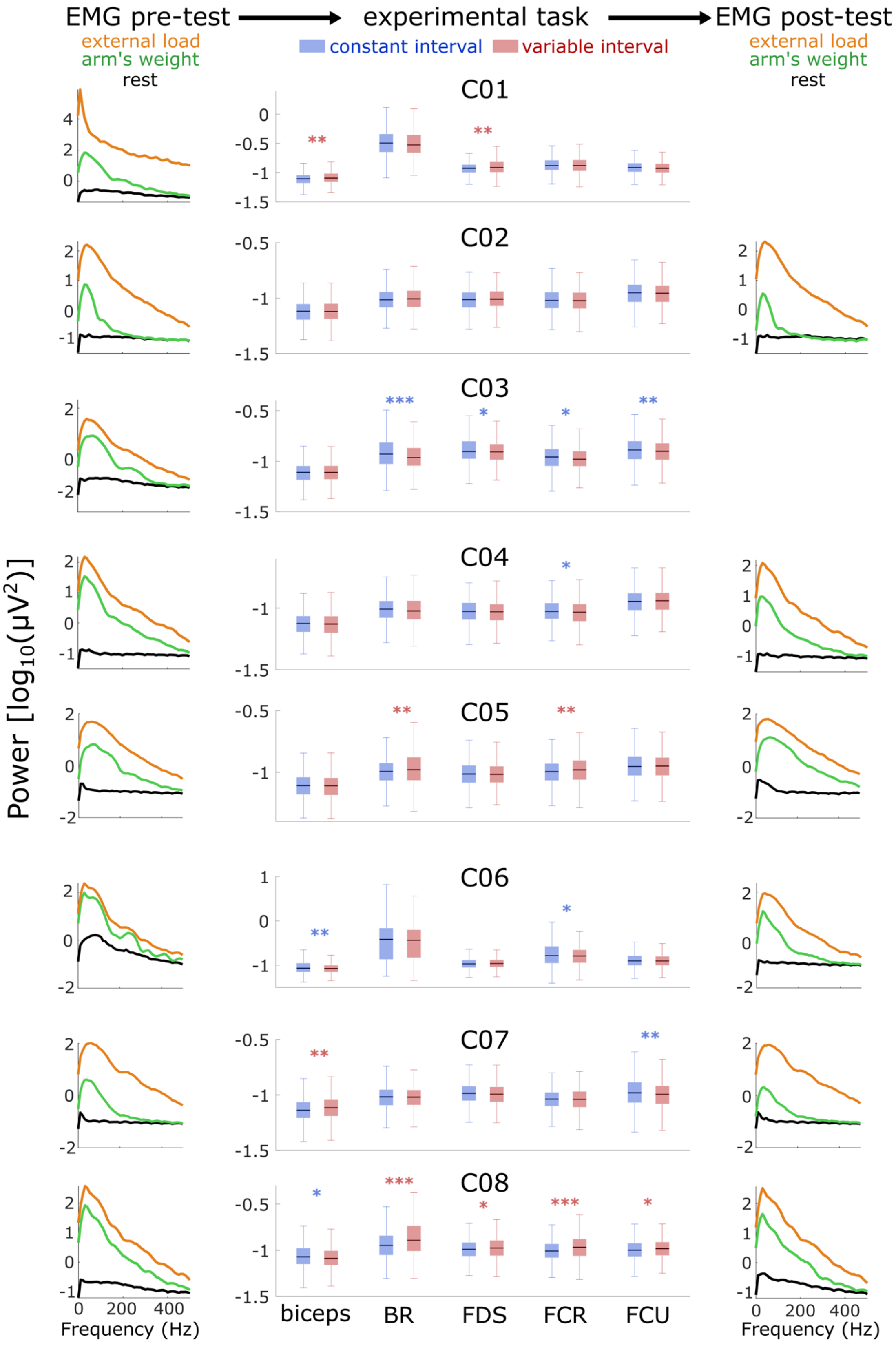

Figure S11.

Temporal predictability of median nerve stimulation does not result in systematic changes to pre-stimulus electromyography (EMG) activity on the stimulated arm. Eight naïve participants (age: 25.63 ± 4.63 years, mean ± SD; five female, seven right-handed)

completed 16 blocks of the experimental task used in the invasive and non-invasive study (Figure 1), in a single session, and under identical conditions as in the non-invasive study (mean median nerve stimulation amplitude: 6.88 mA, range 5.2 to 9.7 mA). Counting accuracy was similar as in the non-invasive study, though the condition difference was not significant (proportion of blocks for which participants counted correctly, mean ± SD: 71.9 ± 31.2 % (constant-interval condition) vs. 60.9 ± 24.5 % (variable-interval condition); $t(7) = 1.5$, $p= .18$). During the task, we recorded EMG activity from five muscles on the left upper arm and forearm via surface electrodes, including the biceps muscle, the brachioradialis muscle (BR), the flexor digitorum superficialis muscle (FDS), the flexor carpi radialis muscle (FCR), and the flexor carpi ulnaris muscle (FCU). For each muscle, we recorded from two electrodes, each placed on the skin above the belly of the respective muscle, with a distance of approximately 2 cm between electrodes (see Supplementary Figure S12A). We used actiCAP slim active electrodes (Brain Products), and recorded at 5000 Hz via an actiCHamp Plus amplifier (Brain Products), with a low-pass filter set to 1350 Hz. We re-referenced offline to a bipolar montage for each muscle, and extracted 100-ms epochs before each median nerve stimulation (to avoid the stimulation artefact, the exact time window was set to -101 to -1 ms before stimulation). We then attenuated line noise at 50 Hz, and harmonics up to 500 Hz, using the same spectrum interpolation approach as in the invasive and non-invasive study. Finally, we computed power between 10 and 500 Hz, in steps of 10 Hz (Hanning taper, 'mtmfft' in FieldTrip). The middle column shows mean power between 30 and 500 Hz for each channel (log-transformed), separately for each participant (C01-C08), and for the constant-interval and variable-interval conditions (blue and red, respectively). Blue asterisks indicate significantly larger EMG activity in the constant-interval condition, compared to the variable-interval condition, while red asterisks indicate the reverse (*, $p<.05$; **, $p<.01$; ***, $p<.001$, uncorrected; Mann-Whitney U test). To ensure that surface EMG was sensitive throughout the experiment, participants completed a pre-test EMG recording before the task (left column), and post-test EMG recording after the task (right column). During the pre- and post-test, we recorded EMG in three conditions. Participants either rested their arm fully on an armrest ("rest" condition, black lines), or held the weight of their unsupported forearm and hand (while the elbow was resting on the armrest; "arm's weight" condition, green), or, additionally, the weight of a sand bag placed on their hand (600 g; "external load" condition, orange). For each condition, we recorded at least 2 minutes, which were cut offline into consecutive 100-ms epochs. The order of conditions ("rest", "arm's weight"', "external load") was counterbalanced between the pre- and post-test EMG session, and across participants. Two participants (C01 and C03) did not complete the post-test EMG recording. While we found preserved sensitivity of EMG recordings throughout the experiment (EMG pre- and post-test) to the weight of the arm and hand, and to the external load, pre-stimulus EMG activity during the counting task was similarly low in the constant-interval and variable-interval conditions, with no systematic condition differences. This indicates that our experimental manipulation of the consistency in the time window between auditory cue and median nerve stimulation did not result in systematic changes to muscle activity on the stimulated arm.

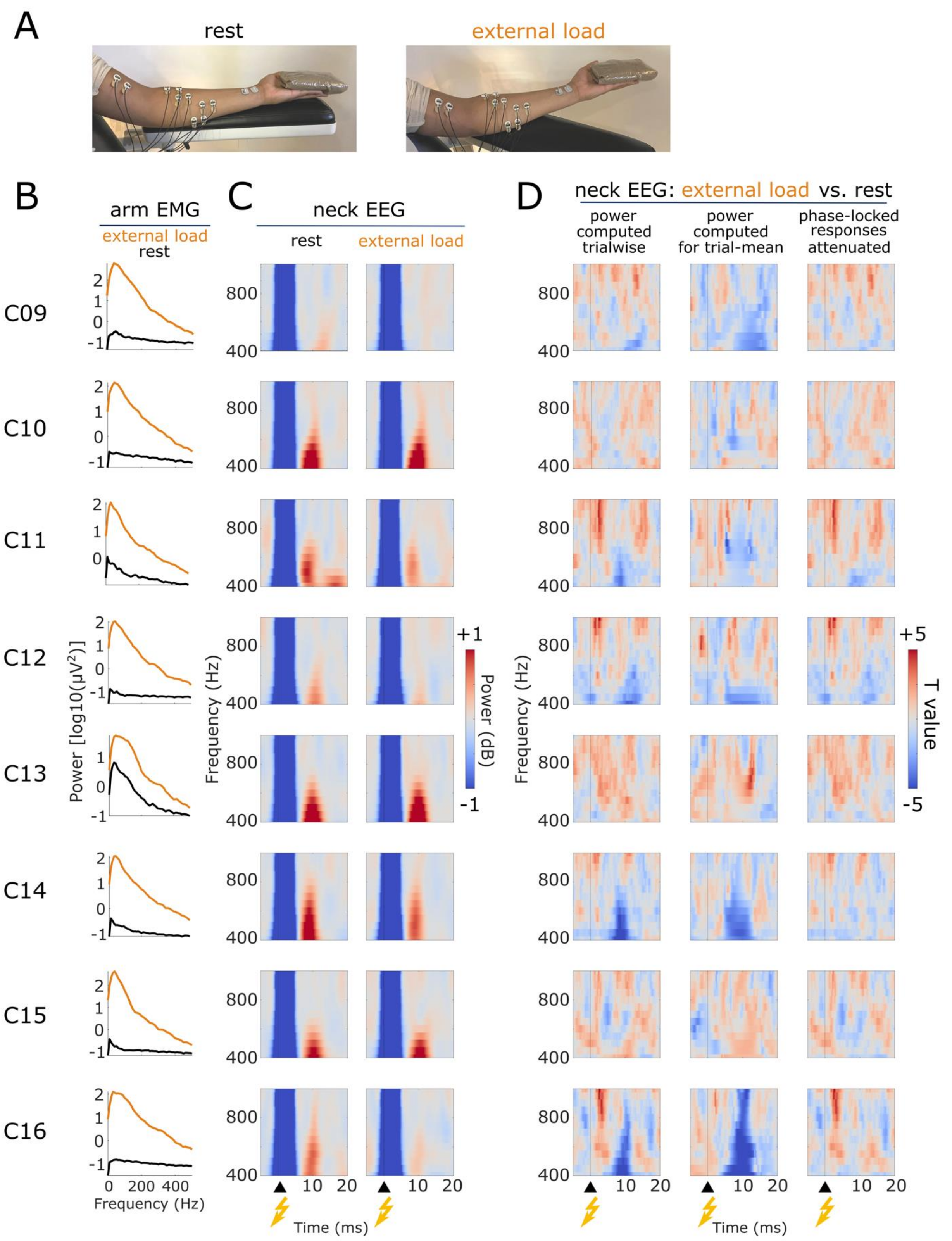

Figure S12.

Voluntary muscle contraction on the arm receiving median nerve stimulation does not reproduce the high-frequency signal modulation by temporal predictability observed in Figures 2 and 3. Eight naïve participants (age: 26.38 ± 6.5 years, mean ± SD; six female, seven right-handed) completed 20 blocks (2 minutes per block) of median nerve stimulation. Stimulation was applied at an average rate of 5 Hz (inter-stimulus interval between 150 and

250 ms, uniform random distribution; mean stimulation amplitude: 5.95 mA, range 5 to 6.5 mA). Each block started with six seconds without median nerve stimulation, allowing us to analyse surface electromyography (EMG) recordings from the stimulated arm in the absence of the electric stimulation artefact. **A**, Participants completed two conditions, alternating by block. In one condition, an armrest fully supported the participants' left forearm and hand, and a sand bag (600 g) was placed on their left hand ("rest" condition). In the other condition, we removed the armrest from underneath the left hand and lower forearm. Participants had to hold the weight of their forearm and hand, and of the sand bag on their hand, in order to maintain the same position as during rest ("external load" condition). This condition thus required strong muscle contraction on the stimulated arm. We recorded short-latency responses to median nerve stimulation from 24 electrodes placed around the neck, following the same montage as in our non-invasive study. In addition, we recorded surface EMG from five muscle on the stimulated arm (see also Supplementary Figure S11). These included the biceps muscle, the brachioradialis muscle, the flexor digitorum superficialis muscle, the flexor carpi radialis muscle, and the flexor carpi ulnaris muscle. For each muscle, we recorded from two electrodes, each placed on the skin above the belly of the respective muscle, with a distance of approximately 2 cm between electrodes. We used actiCAP slim active electrodes (Brain Products), and recorded at 5000 Hz via an actiCHamp Plus amplifier (Brain Products), with a low-pass filter set to 1350 Hz. Reference and ground electrodes were placed on the right and left acromion, respectively. For EMG analysis, the six seconds at the beginning of each block were cut into consecutive segments of 100 ms each, followed by the same EMG analysis approach described in the caption for Supplementary Figure S11. Analysis of data recorded from neck electrodes was identical to data analysis for the non-invasive study (including the same spatial filter; see Methods section). To compare the power of phase-locked responses within individuals, we averaged trials in the time domain per block (i.e., 10 averages per condition and individual) and then transformed trial-averages to the time-frequency domain. We obtained T values by comparing the resulting 10 values per condition in a dependent-samples T test for each time bin and frequency bin, separately for each individual. Attenuation of phase-locked responses followed the same approach described in the Methods section. **B**, EMG activity, averaged across the five EMG channels, was higher in the "external load" condition, compared to the "rest condition". **C**, however, this muscle activity did not reproduce the modulation of short-latency, high-frequency responses that we observed in our invasive and non-invasive study (Figures 2 and 3). Specifically, there was no consistent modulation of high-frequency power across individuals. Importantly, in those individuals, in whom high-frequency power was reduced upon muscle contraction, compared to rest (panel **D**, left column), this effect was clearly bound to phase-locked responses (panel D, middle column), and absent for phase-jittered responses (panel D, right column). This pattern was clearly different from the high-frequency modulation by temporal predictability observed in Figures 2 and 3, which affected phase-jittered responses, but not phase-locked responses. Muscle activity on the stimulated arm can, therefore, not explain the observed short-latency, high-frequency modulation by temporal predictability.

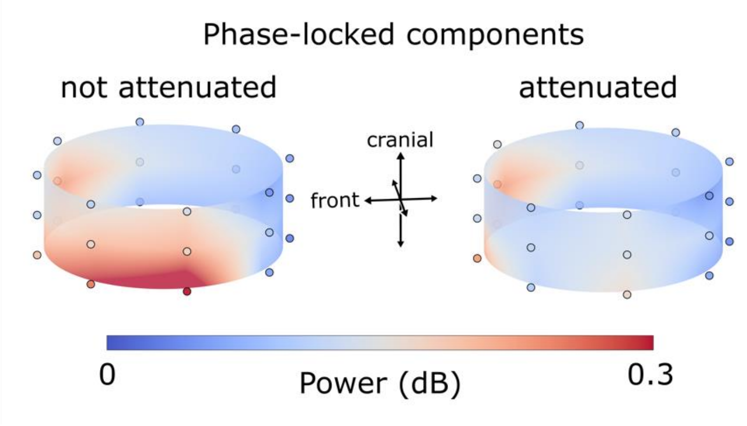

Figure S13.
Attenuating phase-locked responses reduces power primarily in ipsilateral channels. The figure shows topographies of the short-latency, high-frequency power response, collapsed across conditions, and averaged between 400 and 800 Hz, and across the first 16 ms after median nerve stimulation (excluding all time bins possibly affected by the electric stimulation artefact, see semi-opaque area in Figure 3B, C). The left panel shows data without attenuation of phase-locked responses, while the right panel shows data after attenuation of phase-locked responses. The main power difference is in left channels, i.e., ipsilateral to median nerve stimulation.

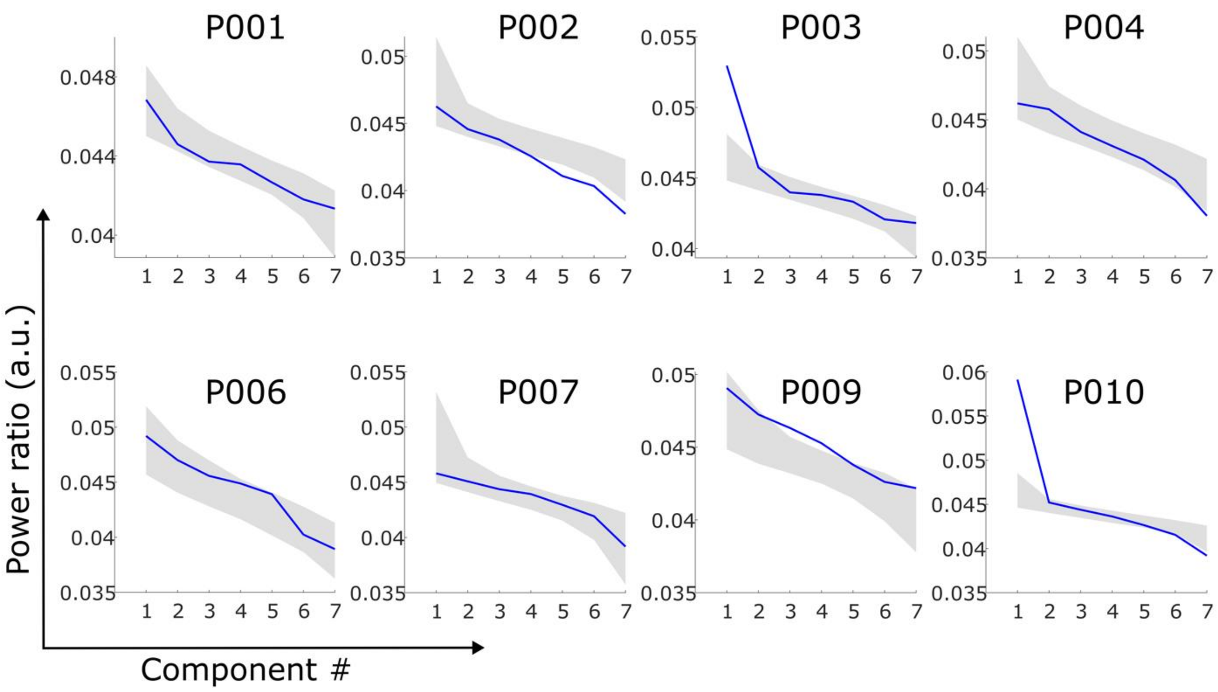

Figure S14.

Power ratios (blue) for a spatial filter set up to emphasize high-frequency responses (200 to 1000 Hz, 8 to 16 ms) in individual patients. The grey shading represents the 5% to 95% percentile range of power ratios obtained from a resampling test under the null hypothesis that any signal in the time-frequency window of interest (200 to 1000 Hz, 8 to 16 ms) is purely noise. Unlike in Supplementary Figure S1B, we observed no sharp decline in power ratios in most patients, so that we could not be sure how many components to choose in order to capture the high-frequency response sufficiently well. Even more importantly, in most patients, the resampling test provided no evidence that any of the components captured more than noise. This contrasts with the clear evidence that a bias filter set up to emphasize the evoked response yielded a component that captured more than noise in most patients (the first component; see Supplementary Figure S1B). One reason why a bias filter set up to emphasize high-frequency responses in our invasive data was not successful could be that the spatial filtering technique we employed relies on an estimation of the covariance matrix, which requires a sufficient signal-to-noise ratio. Compared to the evoked response, the lower-amplitude high-frequency signals may yield a signal-to-noise ratio that is insufficient for a reliable estimation of the covariance matrix at a single-subject level. In our non-invasive study, on the other hand, we could capitalize on the uniform montage across subjects, and the possibility to construct a spatial filter at the group level, enhancing signal-to-noise ratio.

ID	gender	age	pain etiology	medication (CNS active)	stimulation amplitude	initial condition
P001	F	51	CRPS II right hand	Amitriptyline 75 mg, Tilidine 200mg	35 mA	constant
P002	F	33	left C7-Th1 dorsal root trauma	Zolpidem 10mg	35 mA	constant
P003	F	34	left S1 stroke	none	65 mA	variable
P004	F	58	right distal radius fracture	none	65 mA	variable
P006	F	49	nail extraction left hand	Pregabalin 900 mg, Metamizole 500 mg	80 mA	variable
P007	F	40	post-traumatic, left lower arm	Zopiclone 7.5 mg	53 mA	variable
P009	M	64	ischemic stroke right thalamus	Buprenorphine 20µg/h, Metamizole 500 mg, Amitriptyline 25 mg, Venlafaxine 75 mg, Pregabalin 200 mg	60 mA	constant
P010	F	45	post-operative cervico-brachial pain right side (after anterior decompression)	Hydromorphone 24 mg, Metamizole 500 mg	65 mA	variable

Table S1.

Gender, age, clinical data, stimulation amplitude, and starting condition for the patient cohort. CNS, central nervous system.
